# Formulation development of lipid polymer hybrid nanoparticles of doxorubicin and its *in-vitro, in-vivo* and computational evaluation

**DOI:** 10.3389/fphar.2023.1025013

**Published:** 2023-02-07

**Authors:** Muhammad Shafique, Maqsood Ur Rehman, Zul Kamal, Rami M. Alzhrani, Sameer Alshehri, Ali H. Alamri, Mohammed Ali Bakkari, Fahad Y. Sabei, Awaji Y. Safhi, Ahmed M. Mohammed, Mohamed A. El Hamd, Saud Almawash

**Affiliations:** ^1^ Department of Pharmaceutical Sciences, College of Pharmacy, Shaqra University, Shaqra, Saudi Arabia; ^2^ Department of Pharmaceutics, School of Pharmacy, University College London, London, United Kingdom; ^3^ Department of Pharmacy, University of Malakand, Chakdara, (Dir Lower), Pakistan; ^4^ Department of Pharmacy, Shaheed Benazir Bhutto University, Sheringal, (Dir Upper), Pakistan; ^5^ Department of Pharmaceutics and Industrial Pharmacy, College of Pharmacy, Taif University, Taif, Saudi Arabia; ^6^ Department of Pharmaceutics, College of Pharmacy, King Khalid University, Abha, Saudi Arabia; ^7^ Department of Pharmaceutics, College of Pharmacy, Jazan University, Jazan, Saudi Arabia; ^8^ Department of pharmaceutics and pharmaceutical technology Faculty of Pharmacy Al-azhar University, Assiut, Egypt; ^9^ Department of Pharmaceutical Analytical Chemistry, Faculty of Pharmacy, South Valley University, Qena, Egypt

**Keywords:** bioavailability, doxorubicin, lipid polymer hybrid nanoparticles, *in vitro*, *in vivo*

## Abstract

The purpose of this study was to assess the parameters of doxorubicin (DOX) loaded lipid polymer hybrid nanoparticles (LPHNs) formulation development, and then the bioavailability of DOX were determined in the rabbit model, in order to evaluate the intrinsic outcome of dosage form improvement after the oral administration. LPHNs were prepared by combine approach, using both magnetic stirring and probe sonication followed by its characterization in terms of size-distribution (Zeta Size), entrapment efficiency (EE), loading capacity, and the kinetics of DOX. LPHNPs were further characterized by using scanning electron microscopy (SEM), powder X-Ray diffractometry (P-XRD), Fourier transform infrared spectroscopy (FT-IR), differential scanning calorimetry (DSC), *in vitro* and *in vivo* studies. The molecular modeling was determined through the density functional theory (DFT) simulations and interactions. DOX loaded and unloaded LPHNs were administered orally to the rabbits for bioavailability and pharmacokinetic parameters determinations. The plasma concentration of DOX was determined through high performance liquid chromatography (HPLC). The average size of DOX-loaded LPHNs was 121.90 ± 3.0 nm. The drug loading of DOX was 0.391% ± 0.01 of aqueous dispersion, where its encapsulation efficiency was 95.5% ± 1.39. After oral administration of the DOX-LPHNs, the area under the plasma drug concentration-time curve (AUC) improved about 2-folds comparatively (*p* < 0.05). DFT simulations were used to understand the interactions of polymers with different sites of DOX molecule. The larger negative binding energies (−9.33 to −18.53 kcal/mol) of the different complexes evince that the polymers have stronger affinity to bind with the DOX molecule while the negative values shows that the process is spontaneous, and the synthesis of DOX-LPHNs is energetically favorable. It was concluded that DOX-LPHNs provides a promising new formulation that can enhance the oral bioavailability, which have optimized compatibilities and improve the pharmacokinetic of DOX after oral administration.

## 1 Introduction

Globally, cancer causes a major burden of diseases, affecting millions of people out of which half the patients die. A number of chemotherapeutic agents have been modified into various formulations, in order to enhance their therapeutic performances. Doxorubicin (DOX) was the first drug which received clinical approval from the Food and Drug Administration (FDA) as an anticancer drug encapsulated in the form of liposomes DOX belongs to the anthracycline group and is the most commonly used member of this group (Abraham et al., 2005). DOX has been used with common applications in different kinds of cancer including breast cancer, ovarian cancer, lung cancer, and malignant lymphoma (Chan et al., 1999). However, the major side effect of DOX is cardiotoxicity which prevents its long-term use (Lage, 2003; Duggan and Keating, 2011). Besides cardiotoxicity, DOX has some other major shortcomings like poor water solubility, short half-life, gastric instability, and first pass hepatic effects (Peltier et al., 2006; Gou et al., 2011).

Various approaches have been used to improve the oral efficacy of drugs including polymer-prodrugs (PD), polymer-conjugates (PG), polymeric-nanoparticles (PNPs), liposomes, solid lipid nanoparticles (SLNs), etc. (Zhou et al., 2011; Du et al., 2013; Zhang et al., 2015; Ahmad et al., 2018 such as using SLN, layersomes, and dendrimer for DOX (Yang et al., 1999; Ke et al., 2008; Jain et al., 2012), polymeric-micelles for paclitaxel (Yao et al., 2011), and polymeric-nanoparticles for etoposide (Fatma et al., 2016). Poly (lactic-co-glycolic acid) based nanoparticles (PLGA-NPs) were also studied whereby enhancement in gemcitabine bioavailability was observed, (Joshi et al., 2014) in addition, a boosted pharmacodynamics profiles were observed for DOX and paclitaxel (Bhardwaj et al., 2009). Additionally, polyethylene glycol (PEG) containing NPs have been shown to have a more diffusion property with greater penetration properties across the thick layer of mucosa. This adhesive feature leads to enhanced oral bioavailability (Huang et al., 2000; Yoncheva et al., 2007).

DOX loaded to LPHNs can enhanced the oral bioavailability and therapeutic efficacy. Inadequate oral bioavailability is due to the hydrophobic nature of DOX and its poor absorption from duodenal sites (Gold and Moellering, 1996; Walsh, 2000; Huh and Kwon, 2011) and that’s why, it has been categorized as Class-IV based on the Biopharmaceutical Classification System (BCS-IV).

LPHNs were developed as a drug delivery system with characteristic features of both liposomes and polymeric-NPs (Zhang et al., 2008). This hybrid system is a smart drug delivery system with high stability, enhanced entrapment efficiency, attired release kinetics, and fine targeting properties. This study focused at developing physically stable DOX-LPHNs formulation for augmenting its aqueous solubility and enhancing its oral bioavailability, and compatibility. Eudragit RS-100 (polymer), stearic Acid (solid lipid), oleic acid (liquid lipid), and ethyl cellulose (Hepler polymer) were used as excipients in the nano-formulation of LPHNs. The DOX-LPHNs were synthesized and characterized for their surface physico-chemical properties, drug loading, and entrapment efficiencies. The molecular modelling, interaction, and simulations were determined through DFTs. The *in vitro* and *in vivo* release kinetics (plasma concentrations) were determined by various pharmacokinetics models using HPLC, its nano-formulations stabilities and acute toxicities were also determined.

## 2 Materials and methods

### 2.1 Materials

Doxorubicin (Atco Labs Pakistan), stearic acid (SA) (Acros-Organics TFS (United States), Eudragit (Acros-Organics TFS (United State), ethyl cellulose (Acros-Organics TFS (United States), sodium lauryl sulphate (Sigma), and oleic acid (Sigma). All of the solvents used in all the experiments were of analytical grade.

### 2.2 Preparation and optimization of LPHNs (unloaded)

LPHNs were fabricated by a combined process, using both probe sonication and magnetic stirring processes. The unloaded LPHNs were prepared by melting the stearic acid (lipid) at 80°C. The eudragit and sodium lauryl sulphate (SLS) were dissolved in ethanol and were added to the melted stearic acid. The organic phase (ethanol) was removed by stirring while the final volume was adjusted with deionized water. This resultant mixture was passed through probe sonication at 30% amplitude to get LPHNs dispersion. Various approaches regarding the material ratios were adopted for nano-formulations’ optimization, which were compared against the obtained particles size, from LPHNs-1 to LPHNs-6.

### 2.3 Preparation of loaded LPHNs (DOX-LPHNs)

DOX-LPHNs were fabricated by the addition of DOX (40 mg) to the solution of polymer and surfactant in organic phase. Co-encapsulation was carried out by dissolving ethyl cellulose and oleic acid in the same organic solution and the same procedure as were used for LPHNs was followed. The freeze drying or lyophilization was conducted to give stability to LPHNs and DOX-LPHNs and their further conversion to dry powder. Before lyophilization, the samples were subjected to addition of cryoprotectant (glucose solution, 10%) followed by cooling at −20°C overnight. LPHNs/DOX-LPHNs were then transferred to the freeze dryer for lyophilization at temperature of −75°C for about 2 days (48 h), while the increasing temperature was kept around 5°C/h (Abdelwahed et al., 2006).

### 2.4 Entrapment efficiency and drug loading capacity

The optimized formulations of DOX-LPHNs, fabricated by the mentioned technique were centrifuged. The supernatant was separated and further analyzed for un-entrapped drug by UV Visible Spectrophotometer. DOX-LPHNs entrapment efficiency (EE) and drug loading capacity (DLC) for all the prepared samples were calculated by using the following formulae (Ghanshyam et al., 2011; Sadiq and Abdul Rassol, 2014),

$$EE\;\left(\%\right)=\frac{Total\;ammount\;of\;drug\;added-Unloaded\;Drug}{Total\;amount\;of\;drug\;added}\;X\;100$$ (1)

$$DLC\;\left(\%\right)=\frac{Total\;ammount\;of\;drug\;in\;LPHNs}{Amount\;of\;drug\;added+Ammount\;of\;Excipeint}\;X\;100$$ (2)

### 2.5 Surface characterization

#### 2.5.1 Size, zeta-potential and polydispersity index

Size (Z), zeta-potential (ζ), and polydispersity index (PDI) were determined by dynamic light scattering (DLS) technique using the Macrotac Zeta Instruments. To obtain suitable scattering, the LPHNs formulations (both loaded and unloaded) were diluted with deionized water. Measurements were then taken at scattering angle of 90 at room temperature. The particles size, PDI and zeta potential of nanoformulations were calculated by taking the average of three results.

#### 2.5.2 Infrared spectroscopy

IR Prestige 21 Shimadzu (Japan) was used to study the IR Spectra of LPHNs and DOX-LPHNs (Tiţa et al., 2011). During FT-IR studies, scanning was performed at a frequency range of 4,000 cm^−1^ to 450 cm^−1^. For the compatibility of the formulation components, the peaks and patterns shaped by the unprocessed drug were compared with their processed formulations of LPHNs.

#### 2.5.3 Scanning electron microscopy (SEM)

The surface morphology of DOX-LPHNs was studied by using SEM (JEOL, Japan) (Dubes et al., 2003; Uprit et al., 2013). Prior to conducting SEM analysis, deionized water was used to dilute all nanoformulations to form clear and visible samples. Double ended adhesive carbon tape was employed to fixed sample drops of on metallic stub of microscope followed by drying under vacuum for further analysis. Magnification power in the range of 15,000–60000X has been used with varied voltage.

#### 2.5.4 Powder X-Ray diffraction

Powder X-ray diffractometer (JEOL, Japan) was used for unprocessed DOX and processed DOX (DOX-4) to determine changes in the physical state of the drug (Racault et al., 1994). Thus, P-XRD analysis was conducted to study the variations in the crystalline nature and physical state of different samples. P-XRD study was performed using plain plastic holder for sample in the scan range of 2θ = 5–80° with Cu Kα radiation. Tube was operated at 40 kV, 30 mA, step size 0.05°, step time 1.0 s, receiving slit 0.2 mm, scattering slit 1.0° and divergence slit 1.0°.

#### 2.5.5 Differential scanning calorimetry

Thermal analysis of pure DOX, DOX-LPHNs, stearic acid and physical mixture were carried out by DSC (Perkin Elmer-USA). Samples were investigated in aluminum pans at a rate of 10°C/min and DSC thermogram was determined from 40ºC to 400°C (Hou et al., 2003).

#### 2.5.6 Formulation stability studies

Physical stability study was conducted for optimized formulations of LPHNs. The freshly fabricated sample was divided into two parts. Each was then put in two different vials and stored at two different temperatures, i.e., 4°C and 25°C (del Pozo-Rodríguez et al., 2009). After specific intervals of time (1st day, 2nd week, 4thweek, 8th week, and 12th week), both the particle size and Polydispersity index (PDI) were determined through DLS. Data was analyzed statistically by two-tailed *t*-test. Probability ˂0.05 was considered significant.

#### 2.5.7 *In-vitro* release of DOX-LPHNs

Dialysis membrane method was used to study the release of DOX from the DOX-LPHNs polymeric nanoparticles (Bhardwaj and Burgess, 2010). The dialysis membrane soaked in water at least 12 h prior to its use. One ml of DOX-LPHNs (each formulation) was decanted into the dialysis membrane which was then kept at pH 7.4 (50 rpm) using 250 mL of phosphate buffer solution (PBS). We took sample from each formulation after specific time (1–12 h) and analyzed it by means of an UV-spectrophotometer (*λ*_max_ = 278 nm) (Moffat et al., 2011). The release data was tailored into diverse kinetic models to learn both the drug release rate and mechanism of drug release [ (Roohullah et al., 2013), (Taninaka et al., 2000)].

#### 2.5.8 Comparative *in vivo* study

For conducting *in vivo* pharmacokinetic study, healthy rabbits (2 ± 0.3 kg) were used. All experimental animals (rabbits/rats) were screened and accepted for experimental purpose by the Ethical Committee, Department of Pharmacy, University of Malakand (Ref no: UOM/Pharm-IRB-2022/07). The *in-vivo* pharmacokinetic studies were completed in line with the ethical committee of the University of Malakand (Pakistan) and pertinent byelaws, 2008 (Scientific procedure issue 1). All the experimental animals (rabbits) were kept in fasted state (12 h) before dosing but access to water was given. Any experimental animal having dis-comfort was expelled from studies. Prior to oral drug administration, six groups of animals were made, each having *n* = 6 rabbits/group. The optimized LPHNs nanosuspension was administered to Group-I, prepared capsules to Group-II while marketed product to Group-III. At various time interval (0^_^24 h), sample of blood (0.5 mL) was taken from marginal ear vein of rabbits. Blood samples were kept in 3 mL tubes (heparinized), plasma was separated through centrifugation and stored (−20°C) for further analysis.

Different pharmacokinetic parameters were determined for non_−_compartmental model. From concentration_−_time curve, Area Under Curve (AUC_0→t_) was determined *via* trapezoidal rule. From the individual plasma concentration_−_time curve, peak plasma concentration (C_max_) and peak plasma concentration time (t_max_) were calculated. Total area under the curve (AUC_0→24_) was determined by Eq. 3:

$$AUC_{0}\to 24=AUC_{0}\to 24+\frac{C_{t}}{K_{e}}$$ (3)

C_t_ is drug concentration at 24th hour and K_e_ is apparent elimination rate constant.

Relative bioavailability (Fr) after 24 h for equal dose was determined by Eq. 4:

$$Fr=\frac{AUC-LPHNs\;Formulation{\;}_{0\to 24}}{AUC-Marketed\;product_{0\to 24}}$$ (4)

One_−_way analysis of variance and *t*_−_test (*p* < 0.05) were used for statistical analysis of data.

#### 2.5.9 Computational details

The simulations were performed by Gaussian 09 code (Abraham et al., 2005). The Grimme’s dispersion corrected DFT-D3 (Chan et al., 1999; Duggan and Keating, 2011) B3LYP functional was used for all the calculations. The 6-311G (d,p) basis set was applied for geometries optimization. The binding energy (Eb) was calculated using the following equation:

$$Eb=Ecomplex-\left(EM1+EM2\right)$$ (5)

Where, Ecomplex, EM1 and EM2 are the total electronic energies of complex system (polymer bind with doxorubicin drug), monomer-1 (polymer), and monomer-2 (doxorubicin drug), respectively.

#### 2.5.10 Acute toxicity test

Acute toxicity test was based on the chemical testing guidelines of the OECD (Organization for Economic Cooperation for Development) (Jonsson et al., 2013). Mice were used as subject animals which were divided into groups (each *n* = 6). DOX-LPHNs was administered at doses of 50 mg, 100 mg, 200 mg, 400 mg, 800 mg, and 1,600 mg per kg body weight to each group. Morbidity was studied for the first 2 h while mortality was observed post 24 h of dosing. The experimental animals were checked for any behavioral changes as well. The 50% mortality among the rabbits was premeditated by means of the Probit analysis method.

#### 2.5.11 Statistical analysis

All data are presented as the mean ± standard error means (SEM). Statistically significant differences were assessed by one and two-way ANOVA, *t*-test using the graph pad prism software, and the differences were considered significant statistically when *p* < 0.05. Statistical values were indicated in the figure by the following symbols: * indicates *p* < 0.05, ** indicates *p* < 0.01, and *** indicates *p* < 0.001. Probit analysis was used for calculating acute toxicities in the experimental animals for dose-response relationships.

## 3 Results and discussion

### 3.1 Preparation and optimization of LPHNs (unloaded) and DOX-LPHNs (loaded)

**FIGURE 1:**
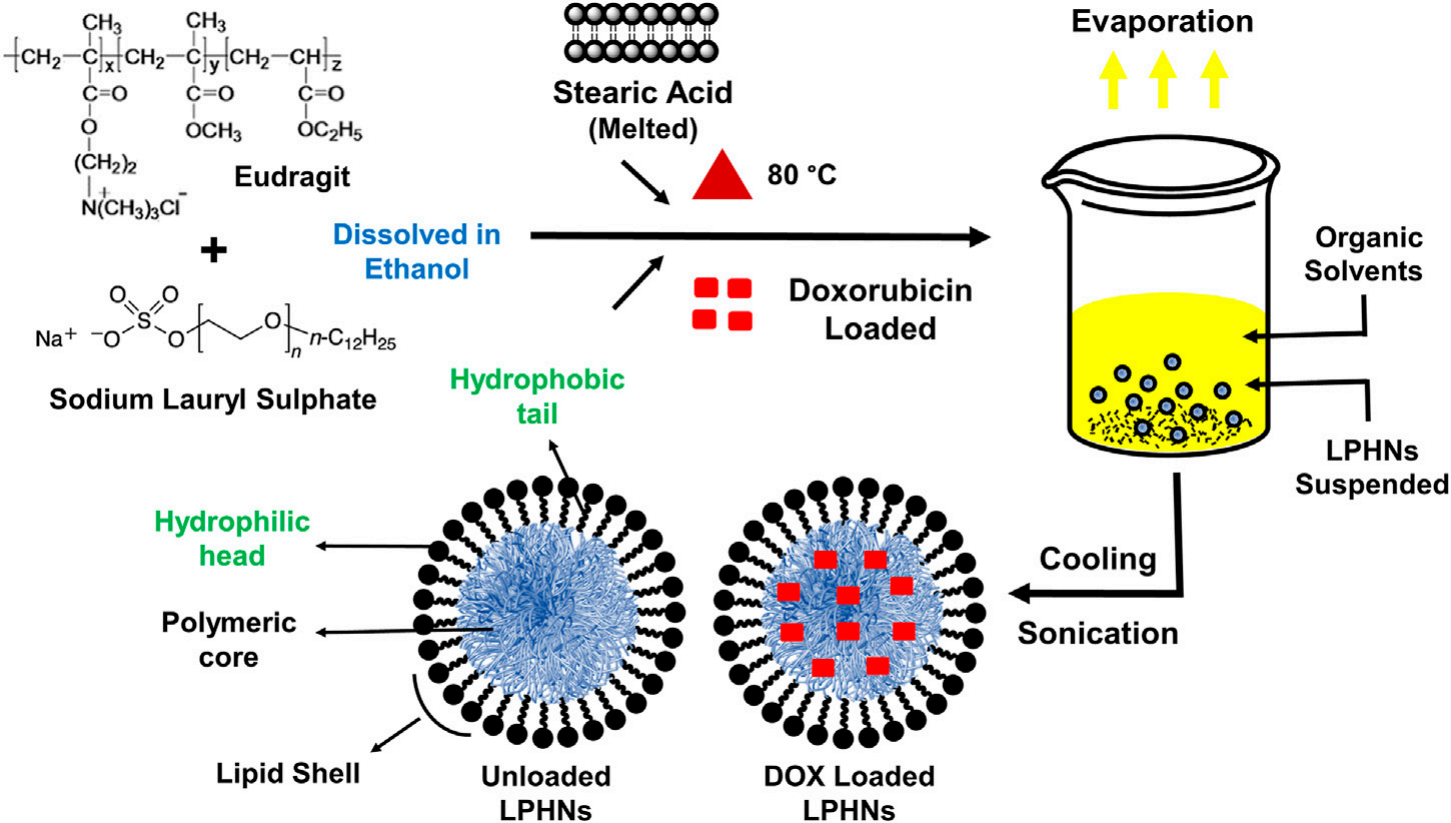
Schematic illustrations showing the preparation of LPHNs (unloaded) and DOX-LPHNs (loaded) formulations.

The detailed schematic illustration of the step-wise procedure for LPHNs and DOX-LPHNs fabrication is shown in Figure 1. The diagrammatical representation of LPHNs is actually combinative approach employing magnetic stirring and probe sonication. For fabrication of LPHNs, stearic acid was used as solid lipid, oleic acid as liquid lipid, sodium lauryl sulphate as surfactant, PEG as co-surfactant, eudragit RS-100 as polymer and ethyl cellulose as helper polymer. Optimization was carried out using different variable factors like concentration of excipients, magnetic stirring and sonication time (Table 1).

**TABLE 1:** Preparation and optimization of unloaded lipid polymer hybrid nanoparticles (LPHNs) nano-formulations.

Formulation code	SA (mg)	Na-LS (mg)	EDG (mg)	Stirring duration (min)	Sonication duration (min)	Particle size ±SEM (nm)
LPHNs-1	500	200	1,000	20	1.5	657.32 ± 5.0
LPHNs-2	500	300	1,000	20	3.0	455.21 ± 4.5
LPHNs-3	500	500	1,000	20	4.5	330.40 ± 5.0
LPHNs-4	500	600	1,000	20	6.0	150.82 ± 4.0
LPHNs-5	500	800	1,000	40	6.0	121.90 ± 3.0
LPHNs-6	500	1,000	1,000	60	6.0	109.25 ± 2.5

SA: stearic acid; Na-LS: sodium lauryl sulphate; EDG: Eudragit. The formulation results were taken in triplicates.

#### 3.1.1 Concentration of surfactant

During optimization of LPHNs sodium laural sulphate is used as surfactant. When the concentration of surfactant was increased, it caused abrupt reduction in particles size. As, further increase in concentration of surfactant showed almost no effect on particle size. It has been reported in literature that higher concentration of surfactant showed lower particle size and also offer better stability to small lipid droplets as it prevent them from coalescence (Kovacevic et al., 2011).

#### 3.1.2 Concentration of co-surfactant

Further decrease in particle size was achieved with addition of co-surfactant. PEG being employed as co-surfactant, further reduced particle size. As, LPHNs fabricated with surfactant/co-surfactant mixture have lower particle size and better stability as compared to LPHNs of unadded co-surfactants.

#### 3.1.3 Stirring time

During variation in magnetic stirring time, reduction in particle size and PDI reduced to the desired acceptable range. During increase in the magnetic stirring time, it has been noticed that particle size also reduced to some extent but it mainly controlled the PDI. Thus, PDI was controlled and reduced by increasing stirring time which has shown almost little bit effect on particle size reduction (Baharifar et al., 2015).

#### 3.1.4 Sonication

During variation in sonication parameters, reduction in particle size was observed. By increasing the sonication time/Hz, particle size reduced to the desired acceptable range. Finally, size was controlled and reduced by sonication which has shown excellent effect on particle size reduction. Important variations in terms of particle size and PDI were seen by changing the mentioned four variable parameters.

**FIGURE 2:**
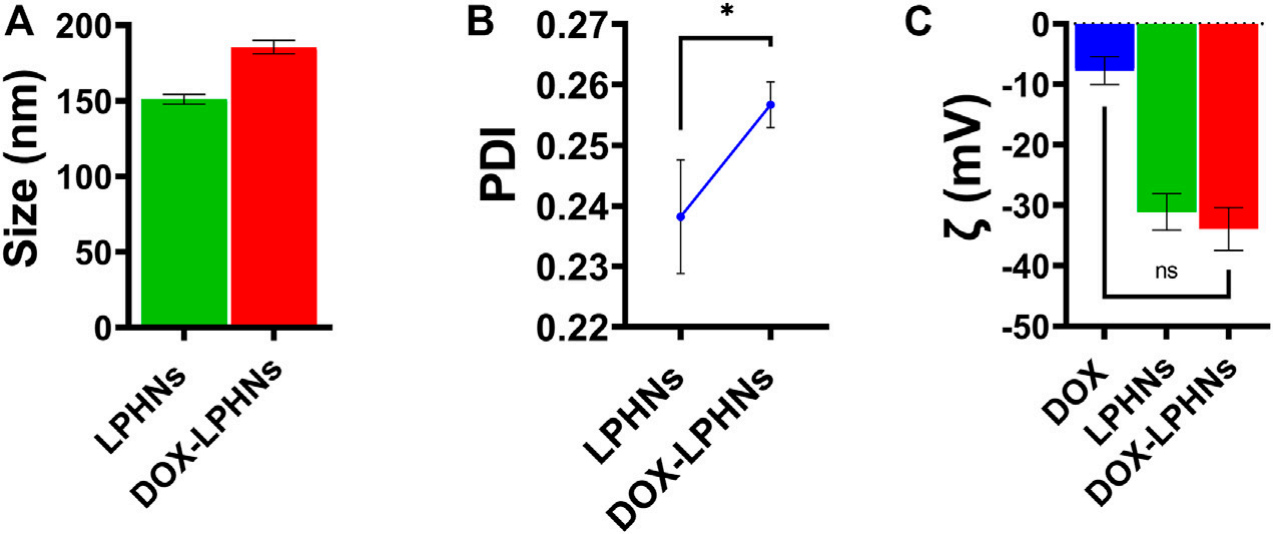
Surface characterization of LPHNs and DOX-LPHNs. **(A)** Average particle size of LPHNs and DOX-LPHNs **(B)** Polydispersity Index of LPHNs and DOX-LPHNs **(C)** Zeta potential (ζ) of LPHNs and DOX-LPHNs. One sample *t*-test (two tailed), *p* value = 0.0999.

During optimization process of blank LPHNs, desired particles size and acceptable PDI were produced with stearic acid, sodium laurel sulphate, PVP and magnetic stirring time (15 min). After optimization of different formulation parameters (concentration of surfactant, magnetic stirring and sonication time) LPHNs and DOX-LPHNs showed optimized average particle size of 150.82 ± 4.37 nm and 185.43 ± 4.43 nm, average PDI of 0.238 ± 0.009 and 0.256 ± 0.003 and zeta potential (ζ) of −31.1 ± 3.0 and −33.95 ± 3.53 respectively (Figure 2). The PDI <0.5 and ZP in the range of ±30 revealed that the fabricated LPHNs would be stable in nature (Ali et al., 2011). For our prepared LPHNs both PDI and ZP were within the acceptable range, which exhibit electrostatic stabilization to avoid aggregation thus preventing particles growth and Ostwald ripening (Liu et al., 2007). As the fabricated loaded LPHNs are for oral administration, so, the produced average particles size is less than 400 nm having the ability of easily crossing the linings of gastro-intestinal cells to achieve the desired boosted oral bioavailability (Suh et al., 2009). Moreover, the fabricated LPHNs comprised of 100–200 nm size range, since particles having size less than 200 nm are undetectable to the Reticulo endothelial system (RES) and remain in circulatory system for a prolonged time period (Bhandari and Kaur, 2013).

### 3.2 Loading capacity and entrapment efficiency

**FIGURE 3:**
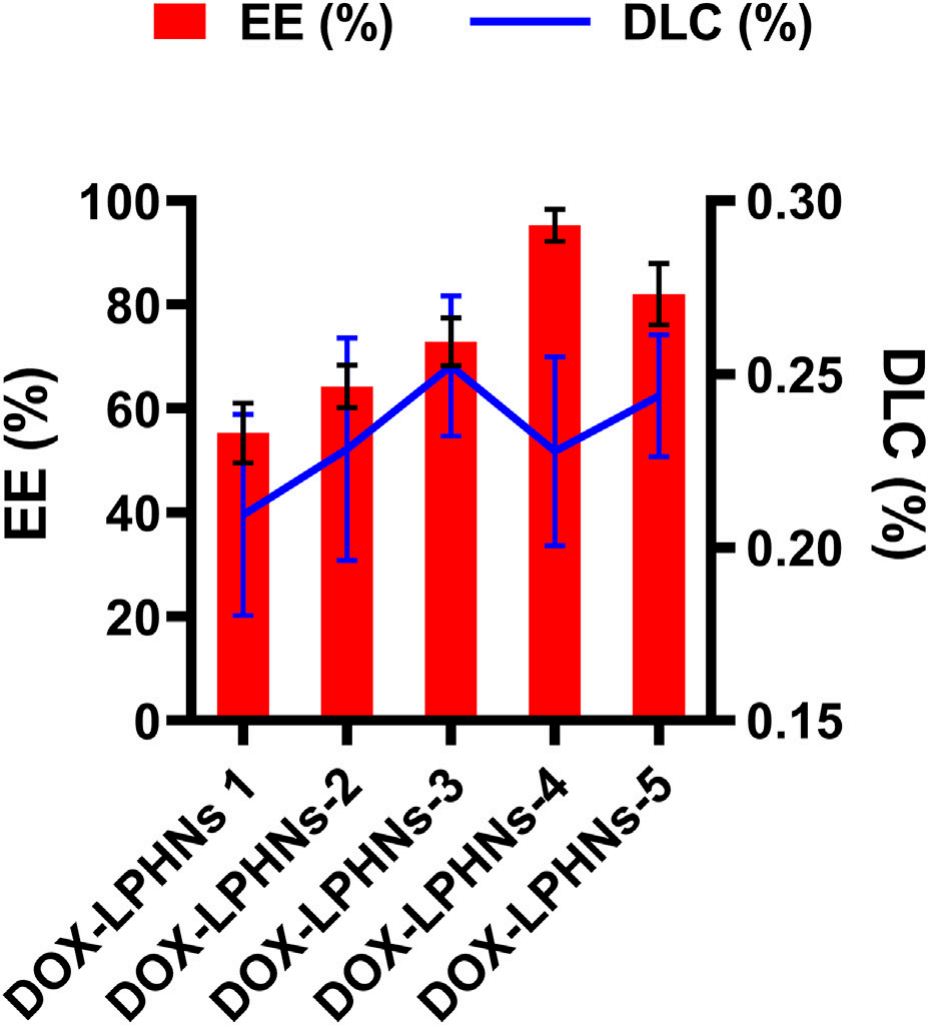
Encapsulation efficiency % (EE) and Drug Loading Contents % (DLC) of doxorubicin loaded lipid polymeric hybrid nanoparticle formulations.

The fabricated DOX-LPHNs were optimized based on the concentrations of oleic acid and ethyl-cellulose to determine EE (%) and DLC (%). All optimized formulations (DOX-LPHNs-1 to DOX-LPHNs-5), along with the concentrations of oleic acid and ethyl-cellulose and EE and DLC are mentioned in Table 2; Figure 3. These results show that addition and then increasing the concentration of oleic acid and ethyl-cellulose increases the EE and DLC significantly.

**TABLE 2:** EE and DLC of different DOX-LPHNs (1–6) formulations with different concentrations.

DOX-LPHNs formulation	Ethyl cellulose (mg)	Oleic acid (mL)	EE±SEM (%)	DLC±SEM (%)
DOX-LPHNs-1	0	0	55.26 ± 5.74	0.209 ± 0.02
DOX-LPHNs-2	0	0.1	64.26 ± 4.10	0.228 ± 0.03
DOX-LPHNs-3	0	0.15	72.86 ± 4.55	0.252 ± 0.02
DOX-LPHNs-4	300	0.2	95.26 ± 3.06	0.227 ± 0.02
DOX-LPHNs-5	500	0.2	82.06 ± 5.93	0.243 ± 0.01

**FIGURE 4:**
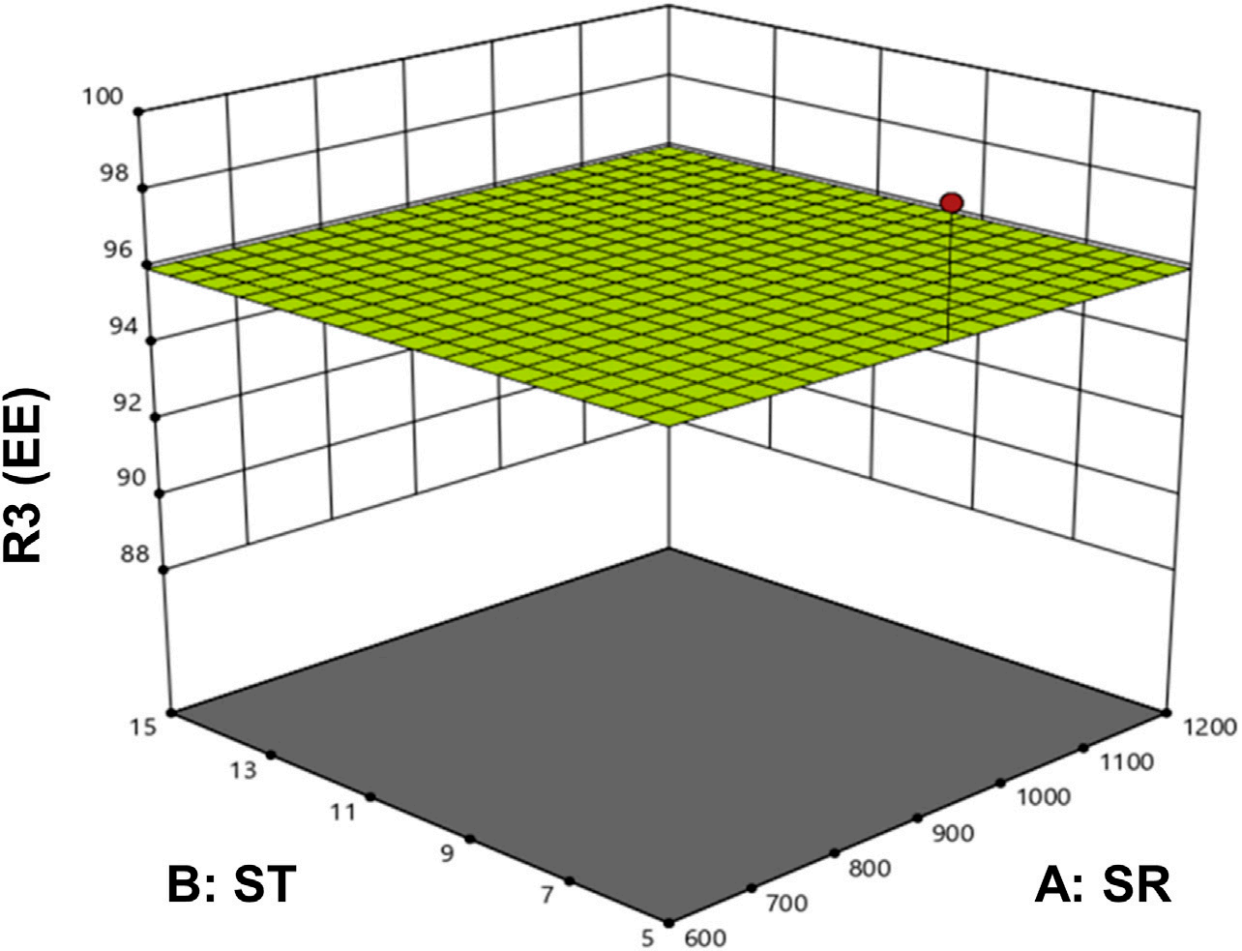
3D Model surface graph for entrapment efficiency (EE).

The optimized formulation of DOX-LPHNs-4 showed 95.26% ± 3.06% for EE and 0.227% ± 0.02% for DLC. Figure 4 shows the 3D model of EE and DLC of DOX-LPHNs.

The combination and specified concentrations of DOX, stearic acid, and polymer were found effective to demonstrate maximum encapsulation of the drug. It has been reported in literature that in polymer and lipid based nano-particulate drug delivery systems, high binding energy of the drugs with the polymers and lipids is required for the successful encapsulation of drugs in polymers as well as lipid layers (Liu et al., 2010). In the reported work, maximum entrapment efficacy and drug loading capacity can be credited to the higher binding energy of the drugs with stearic acid (Liu et al., 2010).

### 3.3 Infrared spectroscopy (drug-excipient’s interaction)

**FIGURE 5:**
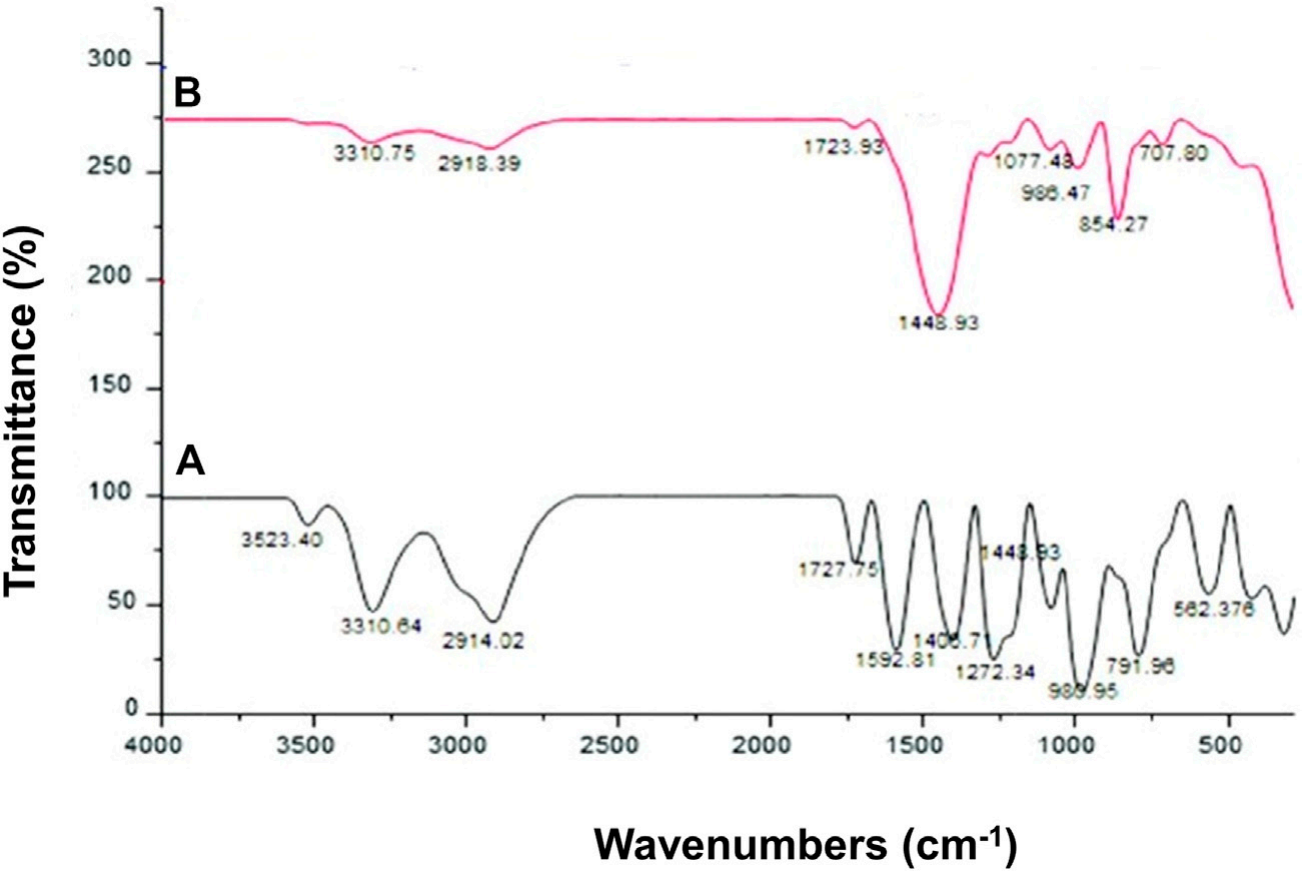
Fourier transform infrared (FT-IR) spectra of **(A)** DOX (Pure) **(B)** DOX-LPHNs.

Figure 5 shows the compatibility of DOX with the formulation components. The peak of –OH in the spectra of DOX-LPHNs has a minor shift to the lower-band and spread to a value of 3,310 cm^−1^. The distinctive peaks at 1,077 cm^−1^, 1,448 cm^−1^, 1723 cm^−1^, and 2,918 cm^−1^ are allocated to carbonyl groups, ketone, and quinone, respectively. The stretching bands of the C–H groups are indicated in peak at 2,918 cm^−1^. The stretching bands of the C=O group(s) are indicated in peak at 1723 cm^−1^. C–C groups stretching bands are at peak of 1,410 cm^−1^. The peak at 1,071 cm^−1^ indicates the stretching bands of the C=O group(s). The minor peak at different places, i.e., 707 cm^−1^, 854 cm^−1^, and 980 cm^−1^ are the stretching bands of the C–O–C groups.

This clearly indicated that the unprocessed samples and their respective prepared loaded LPHNs have similar chemical structure. Thus, no interaction of DOX and excipients was proved by FTIR spectra of unprocessed drugs and processed nanoformulations. This analysis exposed that the formation of a new complex has not been observed among the formulation components, which confirm the compatibility of the drugs with the formulation components. Thus, on the basis of FT-IR analysis, representing no chemical interactions, the prepared loaded nanoparticles can be further processed to achieve the desired boosted oral bioavailability results.

### 3.4 Surface morphology

**FIGURE 6:**
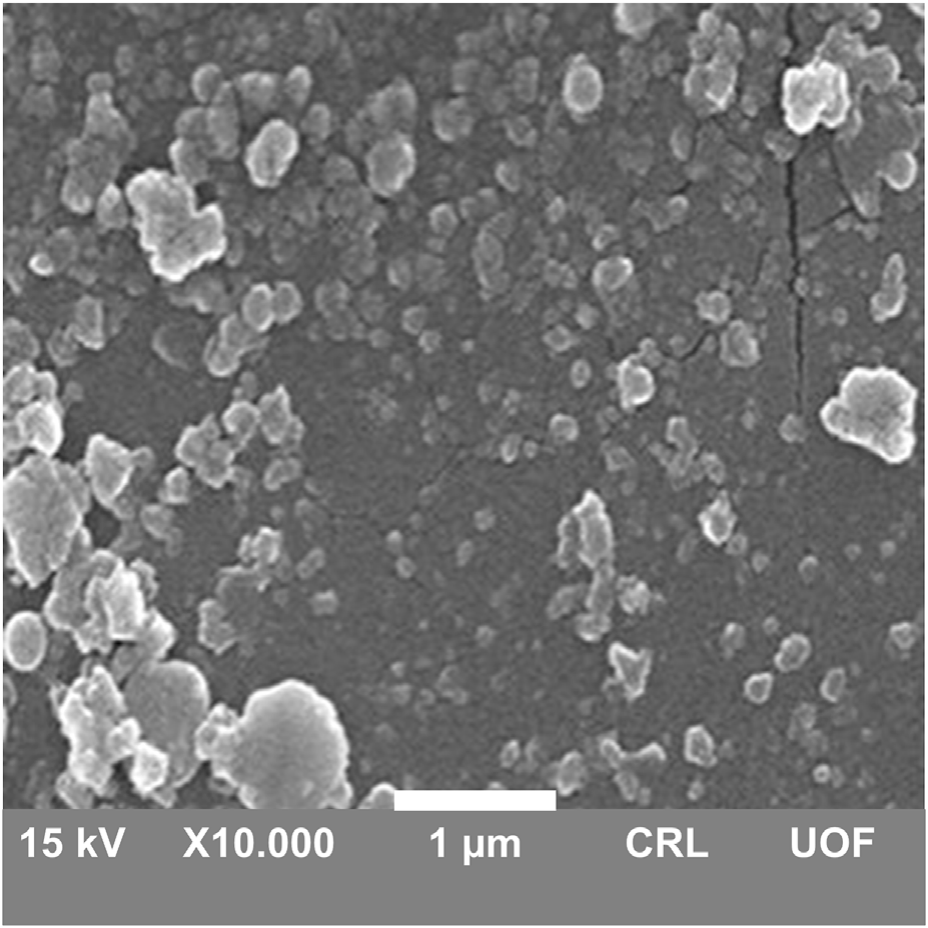
SEM micrograph of DOX-LPHNs.

The surface morphology of DOX-LPHNs was determined by scanning electron microscopy. White patches in micrograph showed solid, identical and fairly spherical shaped nanoparticles with a well-defined periphery (Figure 6). Most of the LPHNs were present in dispersed form with homogeneous distribution which exhibit amorphous nature of the produced nanoparticles. There were some masses of particles which were due to agglomeration. SEM representing nanometric size particles confirmed the results of zeta sizer analysis. Furthermore, the blunt and non-spiky white patches in the micrographs revealed amorphous nature nanoparticles, which plays a vital role in the solubility enhancement of the drugs being a successful outcome of pharmaceutical nanoengineering.

### 3.5 X-ray diffractometry

**FIGURE 7:**
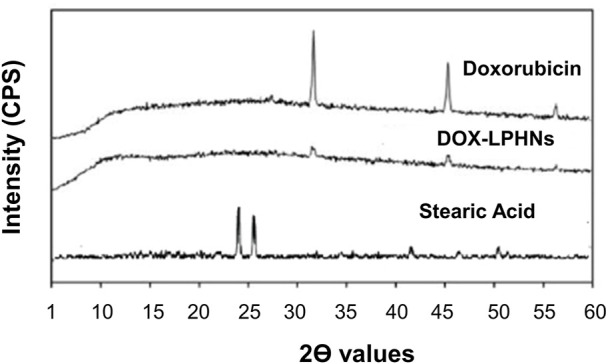
Powder X-RAY Diffraction of DOX, DOX-LPHNs-4, and Stearic acid.

The crystallinity of DOX-LPHNs formulations were determined by powder X-ray diffraction. As shown in Figure 7, the pure DOX had sharp peaks which indicate its crystalline nature while DOX- LPHNs-4 had some diffused peaks which suggests change or decrease in crystallinity of DOX in.

LPHNs formation. Disappearance and reduction in intensities of the peaks in the diffractograms of DOX-LPHNs-4 nanoformulations is indicative for reduction in the crystalline nature (Ali et al., 2011; Khan et al., 2013). Reduction in the crystalline nature to semi-crystalline form or conversion to amorphous form favors increased solubility which in-turn boosted the oral bioavailability (Dang et al., 2009). Semi-crystalline and amorphous drugs have greater free energy compared to crystalline form, so, easily solubilized favoring enhanced oral bioavailability (Müller and Junghanns, 2006; Murdande et al., 2010a; Murdande et al., 2010b; Kakran et al., 2010). Thus, modification in the crystalline nature *via* nano-sizing approach being confirmed by P-XRD studies is highly appreciated and reported in literature (Kakran et al., 2010).

### 3.6 Differential scanning calorimetry

**FIGURE 8:**
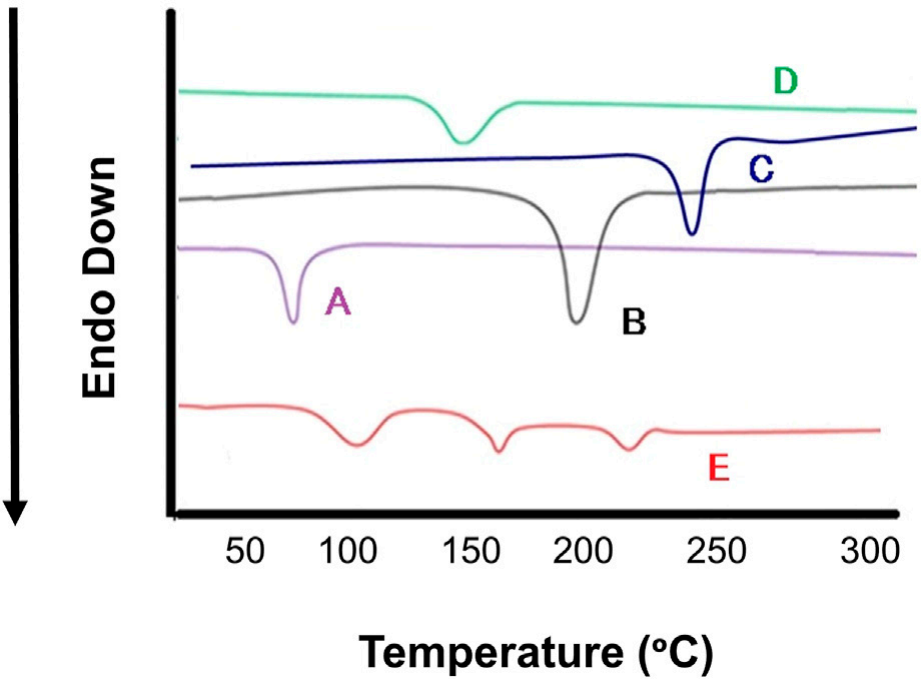
Differential scanning calorimetry (DSC) thermogram of **(A)** Stearic Acid **(B)** Doxorubicin, **(C)** Ethyl Cellulosecellulose, **(D)** Oleic Acid **(E)** DOX-LPHNS-4.

Differential scanning calorimetry was carried out to determine the melting points which further indicates the changes in crystallinity of DOX. DSC study was accomplished for pure DOX, DOX- LPHNs-4, oleic acid, stearic acid and ethyl-cellulose (Figure 8). The mentioned results indicating reduction in particles size, increased surface area as well as closed contact of solid lipid (stearic acid) and polymer with the drug. This change could be considered as a proof for the reduction in the crystallinity of nanoformulations. The mentioned results also showed the dispersion of the drugs in lipid and polymer layers as the level of melting point lowered along with fading of the peaks along with other formulation components. In the literature of LPHNs, the shifting of the melting point peak of drugs to the decreased level has been previously reported (Fang et al., 2008; Farboud et al., 2011).

### 3.7 Stability study

**FIGURE 9:**
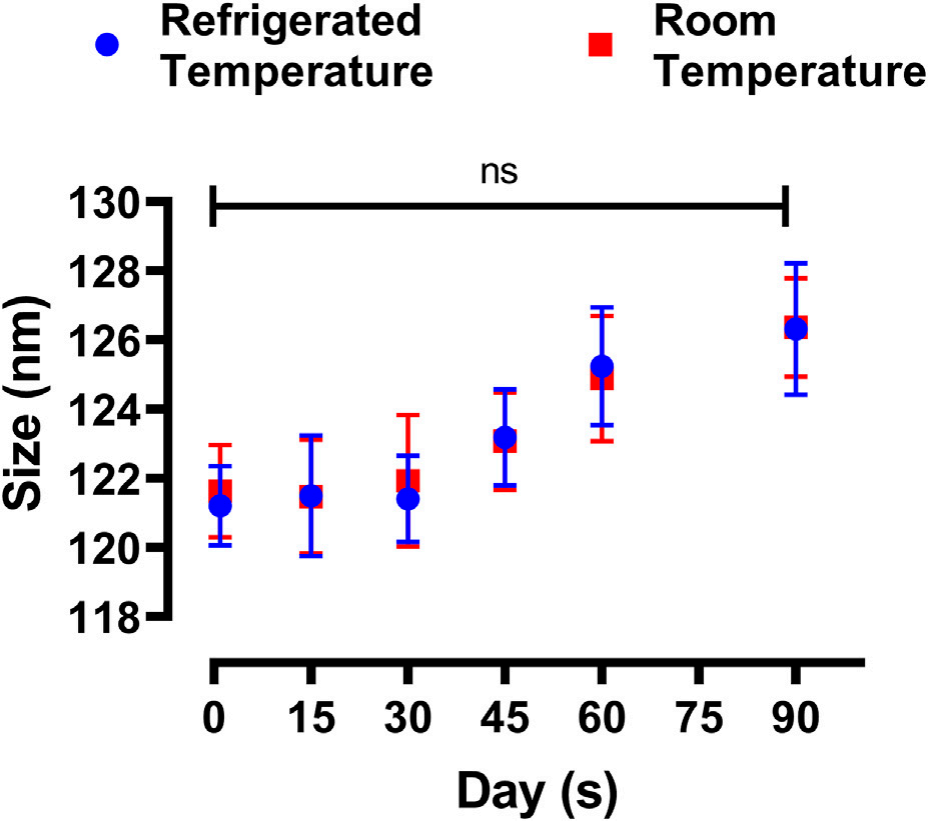
Change in particle size of DOX-LPHNs-4 formulation (DOX-4).

The physical stability of the prepared DOX-LPHNs formulations were assessed both at refrigerated and room temperatures. The Figure 9 shows change in size and PDI of the DOX-LPHNs formulations that were kept for three months, which proposes a long lasting stability of the DOX- LPHNS formulations. At 25°C ± 3.00°C, some rapid growth might be observed for the initial 30 days which may be because of the amorphous nature of the drug loaded LPHNs followed by stabilization for rest of the period. This might be attributed to the dissolution of the small particles while depositing onto the surface of the large particles which is common in amorphous particles (Ali et al., 2011; Khan et al., 2013). Additionally, at room temperature, amorphous solids have increased free energy due to which chemical and physical stability is decreased (Hancock and Zografi, 1997; Khawam and Flanagan, 2006).

### 3.8 *In-vitro* release of the drug

**FIGURE 10:**
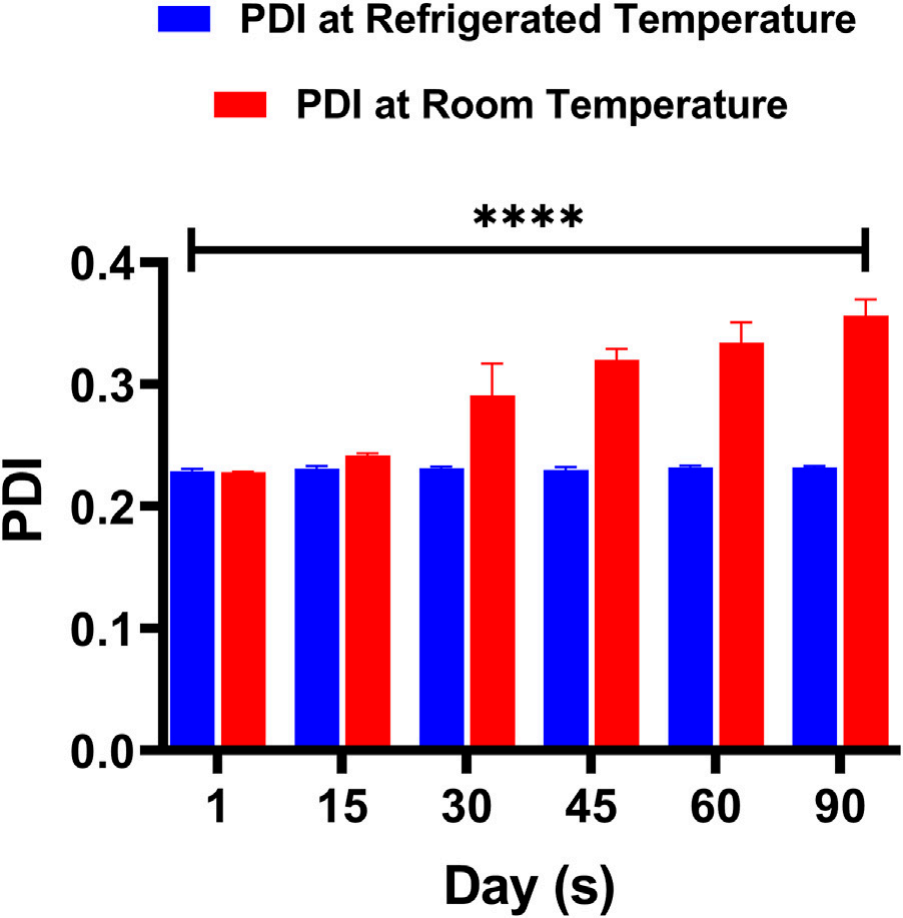
Change in the PDI of DOX-LPHNS-4 formulation (DOX-4).

**FIGURE 11:**
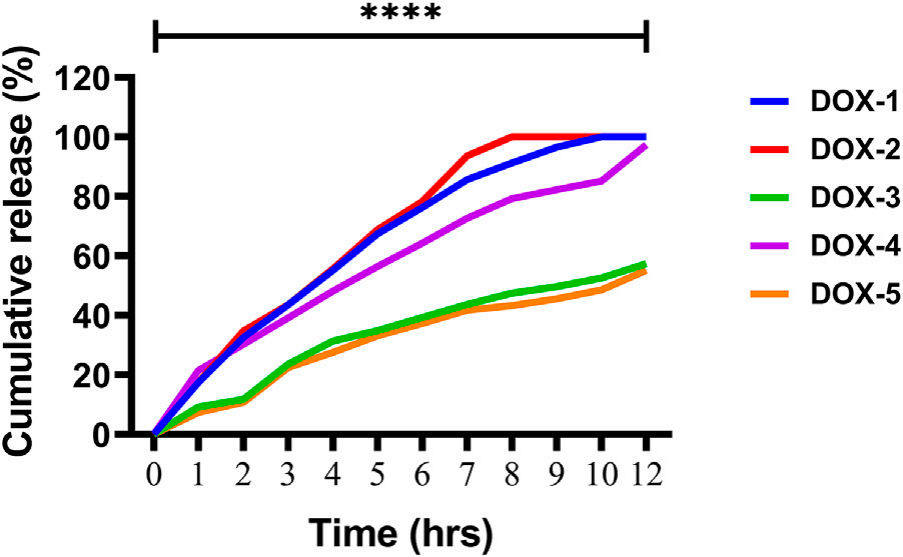
*In-vitro* drug release of the DOX nano-formulations.

The release of DOX from the DOX-LPHNs formulations (DOX-1TO DOX-5) was studied (Zur Mühlen and Mehnert, 1998). It was observed that all nano-formulations (DOX-LPHNs) showed good *in vitro* drug release profile. Initially, burst drug release was observed but later a gradual drug release was observed as shown in Figures 10, 11. This clearly indicated that when drug pay-load increased, cumulative percent drug release decreased and *vice versa*. Thus, it is concluded that increased payload of drugs resulted in prolonged drug release time (Rehman et al., 2015).

### 3.9 Pharmacokinetic modeling and *in vivo* evaluation

The rate and mechanism of DOX release from DOX-LPHNs was studied by putting the release data in different kinetic models. It was observed that the Korsmeyar-Peppas model offered DOX release in the best way. This model showed that the release exponent (n) was more 0.5 which confirmed anomalous transport (Non-Fickian diffusion kinetics) (Barzegar-Jalali et al., 2008; Sadiq and Abdul Rassol, 2014).The *in vitro* drug release rate from LPHNs can be modified on the choice of appropriate surfactant, fabrication variables, polymer concentration and lipid form. A vigorous sustained drug release rate from the polymer hybrid drug delivery system can be provided by the helper polymer and lipid with the optimized concentrations. A stable drug polymer complex lead by compacted interactions between the polymer and drug molecules. Moreover, it leads to a higher sustained drug release profile in contrast with the looser interactions (Ullah et al., 2018). To probe the mechanism of drug release from the hybrid system, various kinetic models were used. It was elucidated that the drug release mechanism from LPHNs has been transformed to anomalous transport (Non-Fickian diffusion kinetics) from diffusion controlled. Dissolution erosion and diffusion is controlling the release of drugs from LPHNs in non-Fickian diffusion kinetics.

**FIGURE 12:**
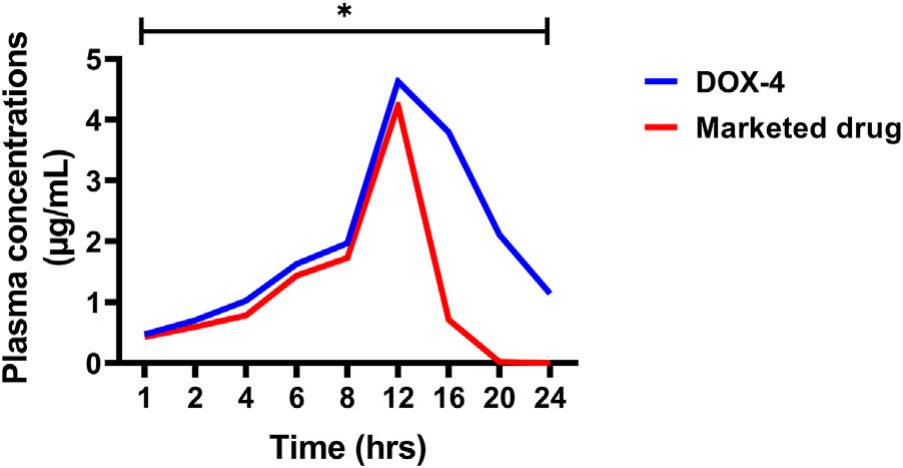
*In-vivo* pharmacokinetic profile of doxorubicin (DOX-4) and marketed DOX.

The *in vivo* pharmacokinetic parameters of DOX-LPHNs and marketed DOX, i.e., C_max_, T_max_ AUC, and t_1/2_ are present in Tables 3, 4, while the Figure 12 shows comparative *in-vivo* release of drug from DOX-LPHNs and marketed DOX. The plot shows the plasma concentration vs*.* time curve. The data obtained from this study as compared to DOX-treated rabbits at the respective time-period are shown here as mean ± SEM (^*^ *p* < 0.05, ^**^ *p* < 0.01, ^***^ *p* < 0.00). The data was statistically significant after two-way analysis and post-hoc Bonferroni’s analysis. DOX loaded LPHNs at a dose of 20 mg/kg body weight showed higher C_max_ (3.333 μg/mL) as compared to the marketed drug (1.65 μg/mL). Similarly, the t_max_ for DOX loaded LPHNs was observed as 0.31 h while for marketed DOX as 0.634 h. Similarly, the t_1/2_ for marketed drug was 9.14 h and for DOX loaded LPHNs was 26.07 h. The area under concentration-time curve from time zero to 24 h for DOX loaded LPHNs was 33.23 μg h/mL while for marketed DOX was 17.20 μg h/mL. Optimized DOX loaded LPHNs showed considerable variations in the pharmacokinetics of DOX. A notable rise in the peak plasma concentration (C_max_) and elimination half-life (t_1/2_) with a significant drop-in time required for peak plasma concentration in comparison with marketed DOX. Correspondingly, the DOX loaded LPHNs (DOX-4) also showed enhancement bioavailability of DOX. Area under concentration time curve (AUC) for the marketed doxorubicin decreased by 50% as compared to DOX LPHNs (DOX-4) in the bloodstream after oral administration (Zhang et al., 2012).

**TABLE 3:** Pharmacokinetic modelling of the DOX-LPHNS formulations.

Formulation	Zero order (*R*2)	First order (*R*2)	Higuchi model (*R*2)	Korsmeyar-peppas model	
				(n) (*R*2)	
DOX-1	0.921	0.872	0.934	0.676,583	0.947
DOX-2	0.937	0.967	0.976	0.778,234	0.949
DOX-3	0.946	0.947	0.967	0.812,346	0.955
DOX-4	0.973	0.984	0.990	0.865,728	0.960
DOX-5	0.988	0.957	0.971	0.962,093	0.971

**TABLE 4:** Pharmacokinetic parameters of Doxorubicin (DOX-4) and marketed DOX.

Sample	Pharmacokinetic parameters of doxorubicin (DOX-4) and marketed DOX			
	*T*_*1/2*_ (hrs)	*T*_max_ (hrs)	*C*_max_ (µg/mL)	AUC_0t_ (µg/mL)
Doxorubicin (DOX-4)	26.07 ± 3.273***	0.31 ± 0.874**	3.333 ± 0.2963**	33.23 ± 4.486**
Marketed DOX	9.14 ± 1.21*	0.634 ± 1.042	1.658 ± 0.2.124	17.20 ± 3.218*

### 3.10 Computational analysis

**FIGURE 13:**
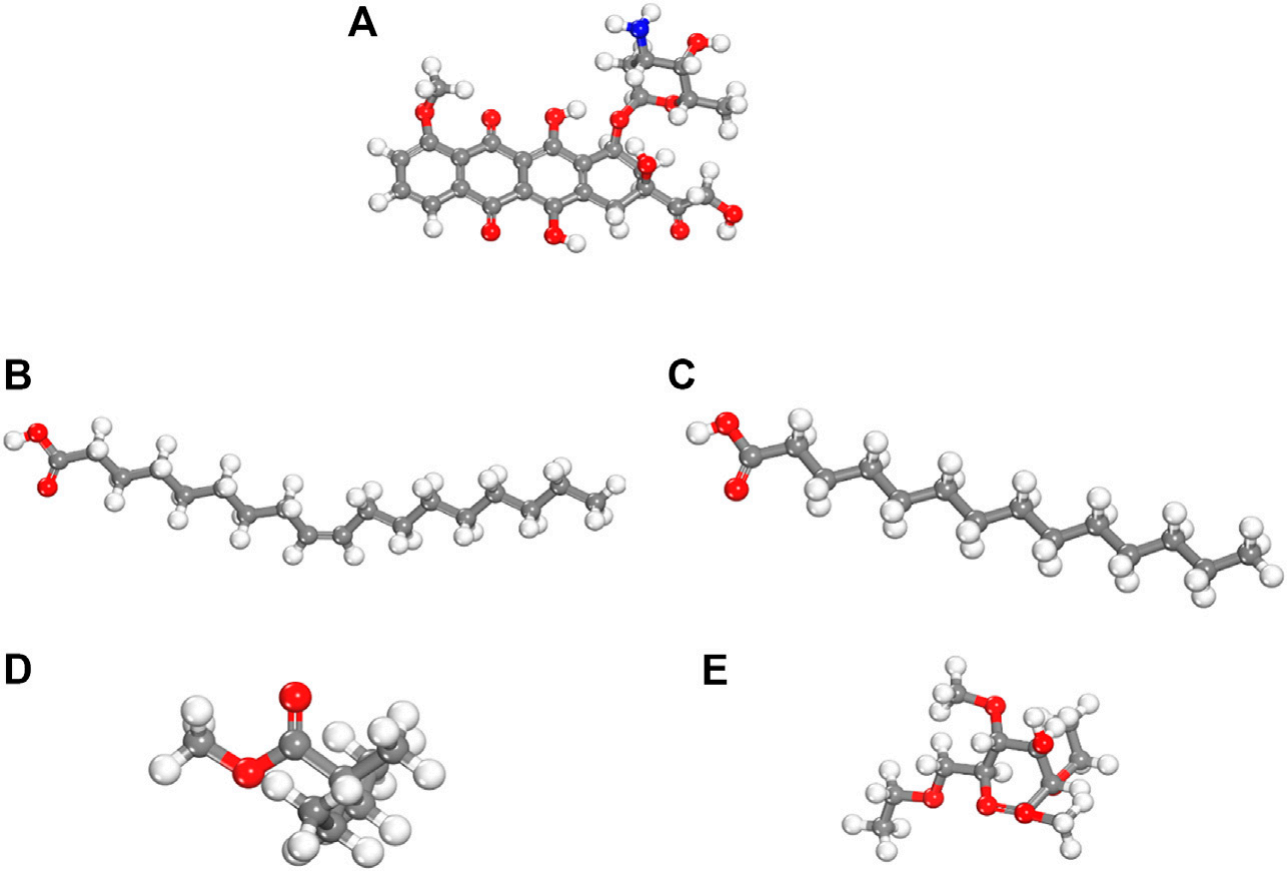
dft optimized geometry of **(A)** dd molecule **(B)** oa **(C)** sa **(D)** ec and **(E)** ers-100. White, grey, and red balls represent hydrogen, carbon, and oxygen atoms, respectively.

**FIGURE 14:**
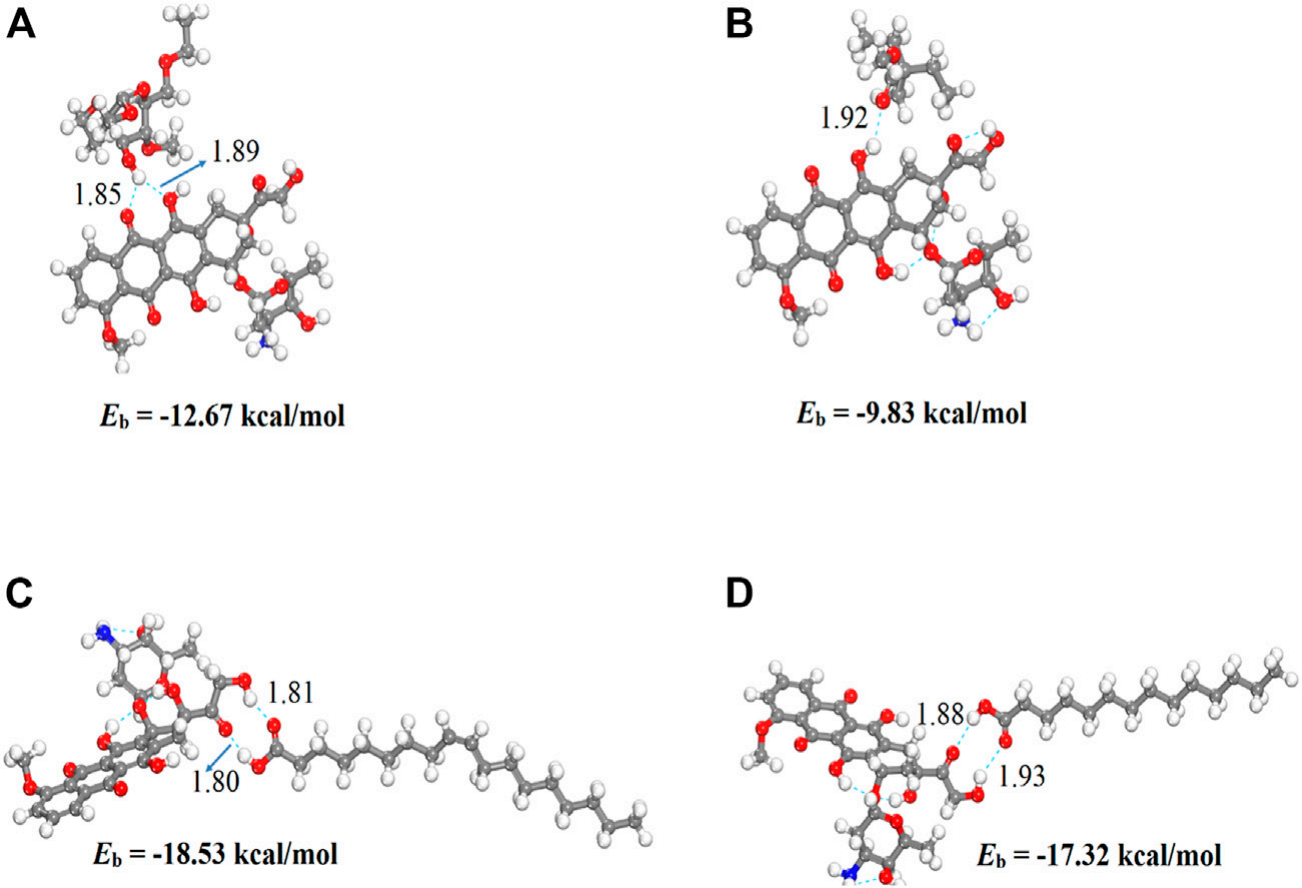
Optimized structures of DD complexes with polymers unit **(A)** EC/DD **(B)** ERS-100/DD **(C)** OA/DD AND **(D)** SA/DD. Bond distances are in Å.

We performed density functional theory (DFT) simulations to gain deeper insights in the interaction mechanism of the drug molecule with the polymers. Before simulating the drug interaction mechanism, initially, we optimized the geometries of the monomers (drug and polymers) system, to understand the reactive sites in the monomers system, which will interact during the reaction process. The optimized geometries of the monomers are illustrated in Figure 13. The structure of doxorubicin drug, Eudragit RS-100 (polymer), stearic Acid (solid lipid), oleic acid (liquid lipid), and ethyl cellulose (Hepler polymer) are represented by DD, ERS-100, SA, OA, and EC, respectively. The white, grey, blue, and red balls in the structures show the hydrogen, carbon, nitrogen, and oxygen atoms, respectively. The values of Mullikan atomic charges extracted from the geometries of monomers are listed in Table 5. The Mullikan atomic charges for nitrogen (−0.61 e) and oxygen atoms (−0.46 to −0.51 e) of both DD and polymers are highly negative while the values of hydrogen atoms as strongly positive. This shows that the O and N atoms are strong nucleophilic sites, and the hydrogen atoms are the electrophilic sites. Thus, we made different complexes, where the nucleophilic and electrophilic sites of DD and polymers are directed to each other and full geometry relaxation were performed. Thus, on the basis of the atomic charges, we interacted the DD with the EC unit of polymer *via* C=O and OH sites with OH site of EC represented in Figure 14A. The values of intermolecular bond distances, adsorption energies and charge transfer are given in Table 5. The geometry relaxation evinces that the polymer (EC) makes between two intermolecular hydrogen bonds with the DD *via* its OH group, i.e., OH---OH with bond distance of 1.89 Å and OH---OC with bond distance of 1.85 Å, respectively. The binding energy (Eb) value obtained for this complex was −12.67 kcal/mol. The charge transfer analysis showed that −0.021 e is transferred to the O atom from H in OH---OH bond and −0.044 e in OH---OC bond. While in complex-2 (Figure 14B, the ERS-100 polymer interacted with the DD through the OH site. In this complex, only one intermolecular hydrogen-bond (H-bond) with the OH group of the DD formed. The bond length value calculated for OH---OH H-bond was 1.92 Å and the Eb value obtained was −9.83 kcal/mol, while −0.043 e is transferred from the O atom to the H atom after complexation. Moreover, in complex-3 (Figure 14C, the COOH group OA is directed towards the OH and CO sites of DD. The complex-3 shows two H-bonds with the COOH group of the OA, i.e., OH---OC having bond length of 1.80 Å, on the other hand the bond length of CO----HO is 1.81 Å which is almost similar in strength with the OH---OC bond. The shorted H-bond formation depicts the stronger electrostatic interaction between the OA polymer and DD. This leads to the larger *E*_b_ value of −18.53 kcal/mol, which is larger compared to the other complexes. Due to stronger electrostatic interaction, the charge transfer observed between the H and O atom was very large (−0.059 and −0.057 e). Similarly, in complex-4, the COOH group interacted with the C=O and O—H sites of DD. The geometry relaxation evinces the formation of two stronger H-bond formation with binding distance of 1.88 Å and 1.93 Å, which results in *E*_b_ value of −17.32 kcal/mol while the charge transferred value noticed was −0.052 and −0.049 e. Thus, the DFT simulations showed that the polymers molecules have greater affinity to bind with the DD molecule and the binding distances, binding energies and charge transferred results demonstrated that the synthesis of DOX-LPHNs is strongly favorable.

**TABLE 5:** Bond distances (Å), binding energy (*E*_b_ kcal/mol) and charge transferred (Q_CT_ e) for the different complexes of DD with polymers obtained though DFT simulations.

Complexes	Bond distance	*E*_b_	Q_CT_
EC/DD	1.85, 1.89	−12.67	−0.044, −0.021
ERS-100/DD	1.92	−9.83	−0.043
OA/DD	1.81, 1.80	−18.53	−0.059, −0.057 e
SA/DD	1.88, 1.93	−17.83	−0.052, −0.049

### 3.11 Toxicity study

No behavioral change was observed in the first 2 h after administration of DOX-loaded LPHNs. Similarly, post 24 h of DOX-loaded LPHNs administration (50 mg/kg to 400 mg/kg), no death was noted. When the dose of the drug was increase from 400 mg/kg to 800 mg/kg, 16% death (mortality) rate was observed. In the same pattern, when experimental dose was increased to 1,600 mg/kg, the mortality rate almost doubled to 32.6%. This study revealed that acute toxicity or LD_50_ for DOX loaded LPHNs is more than 1,600 mg/kg (Table 6).

**TABLE 6:** Acute toxicity test of DOX nanoparticles.

Dose (mg/kg)	No. of deaths	Percent deadliness	LD_50_ (mg/kg)
50	0	00	>1,600
150	0	00	
300	0	00	
400	0	00	
800	1	16	
1,600	2	33.5	

*n* = 6 Mice per dose group from the acute toxicity study, it was concluded that the percent mortality was 16.6% with a dose of 800 mg/kg. The LD_50_ value for DOX, nanoparticles was higher than 1,600 mg/kg.

## 4 Conclusion

It is proved that DOX loaded in lipid polymer hybrid nanoparticles (LPHNs) is a good nanomedicine having the desired value_−_added characteristics. Similarly, it has been shown that DOX and excipients have an excellent interaction as well affinity. LPHNs were fabricated *via* combinative approach of magnetic stirring and sonication. No sophisticated apparatus was used during the fabrication procedure. Surfactant and co-surfactant were used during LPHNs fabrication to stabilize the developed formulation. They were more stable at cold temperature (5°C ± 3.00°C). The developed formulation was easy, simple, and reproducible with the potential to easily scale up for large scale production. *In vitro* and *in vivo* studies confirmed sustained drug release behavior and improved bioavailability. The DFT calculations demonstrated that the polymers have stronger affinity towards the DD molecule. The polymers interacted with different sites of the DD through stronger electrostatic intermolecular interactions with shorter bond distances. In addition, the larger negative binding energies (−9.83 to −18.53 kcal/mol) showed that the interaction mechanism is spontaneous, and the polymers have greater affinity to stably deliver the DD molecule. Thus, it is concluded that we successfully prepared LPHNs loaded with DOX showing sustained release.Thus, it can be concluded that an attempt can be made to produce DOX loaded in lipid polymer hybrid nanoparticles (LPHNs) which could potentially be converted into a suitable solid dosage form followed by its comparative *in vitro* and *in vivo* assessments.

## Data Availability

The original contributions presented in the study are included in the article/Supplementary Material further inquiries can be directed to the corresponding author.
