# Supplementary figures and images for: Formulation development of lipid polymer hybrid nanoparticles of doxorubicin and its in-vitro, in-vivo and computational evaluation
Source: Front Pharmacol. 2023 Feb 7;14:1025013. doi: 10.3389/fphar.2023.1025013 (PMC9941671; doi:10.3389/fphar.2023.1025013)

## Slide 1
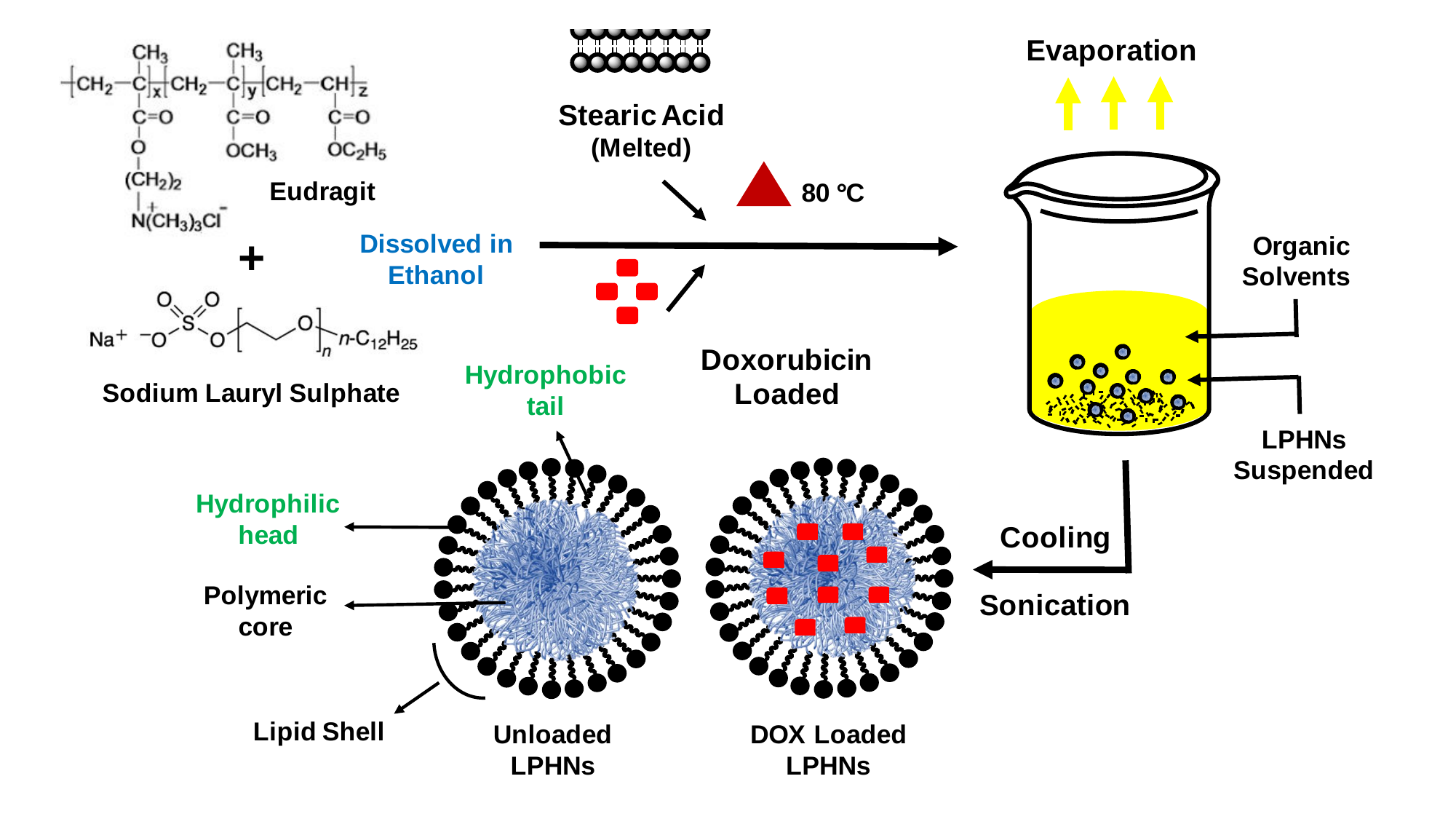

## Slide 2
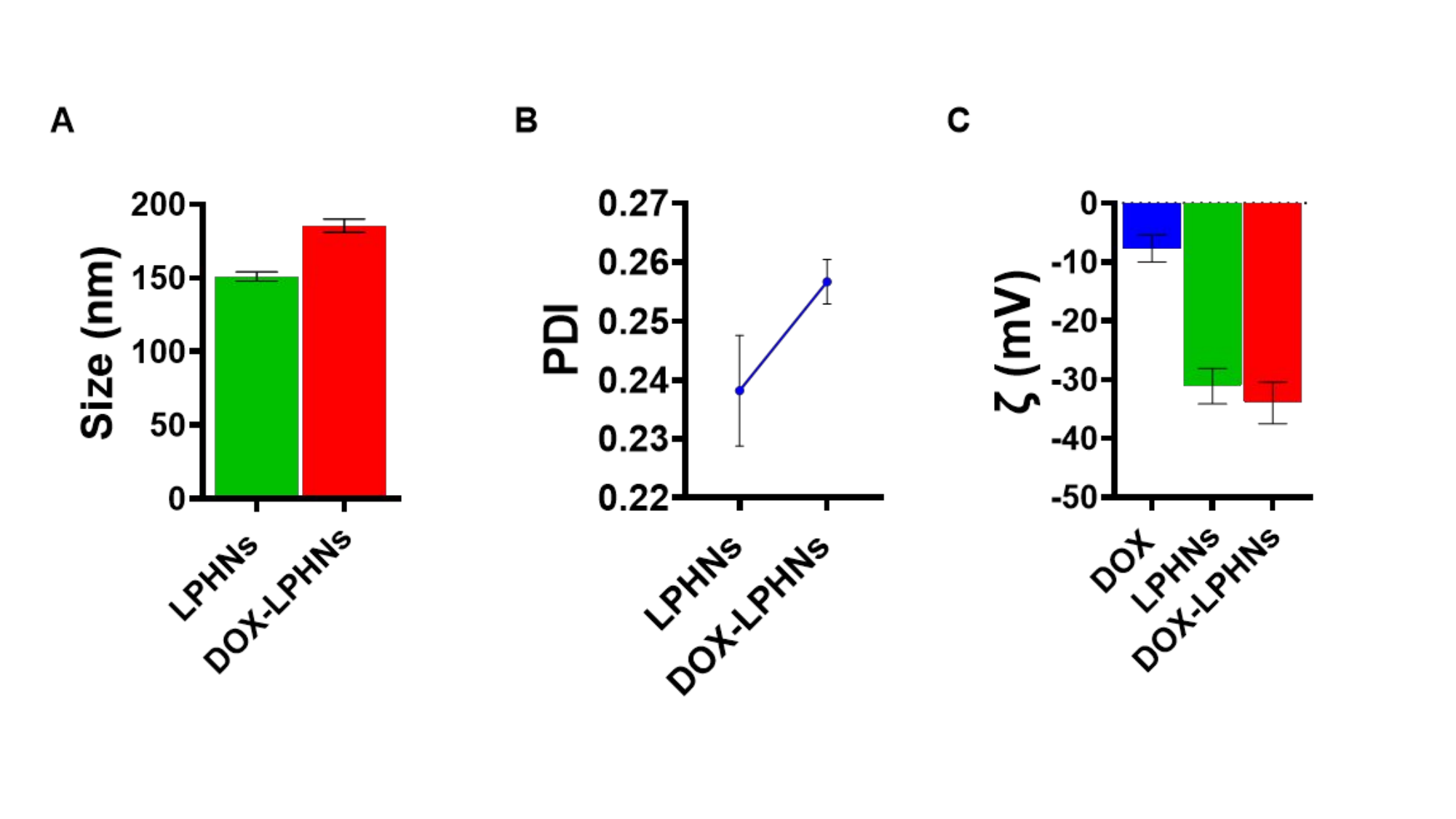

## Slide 3
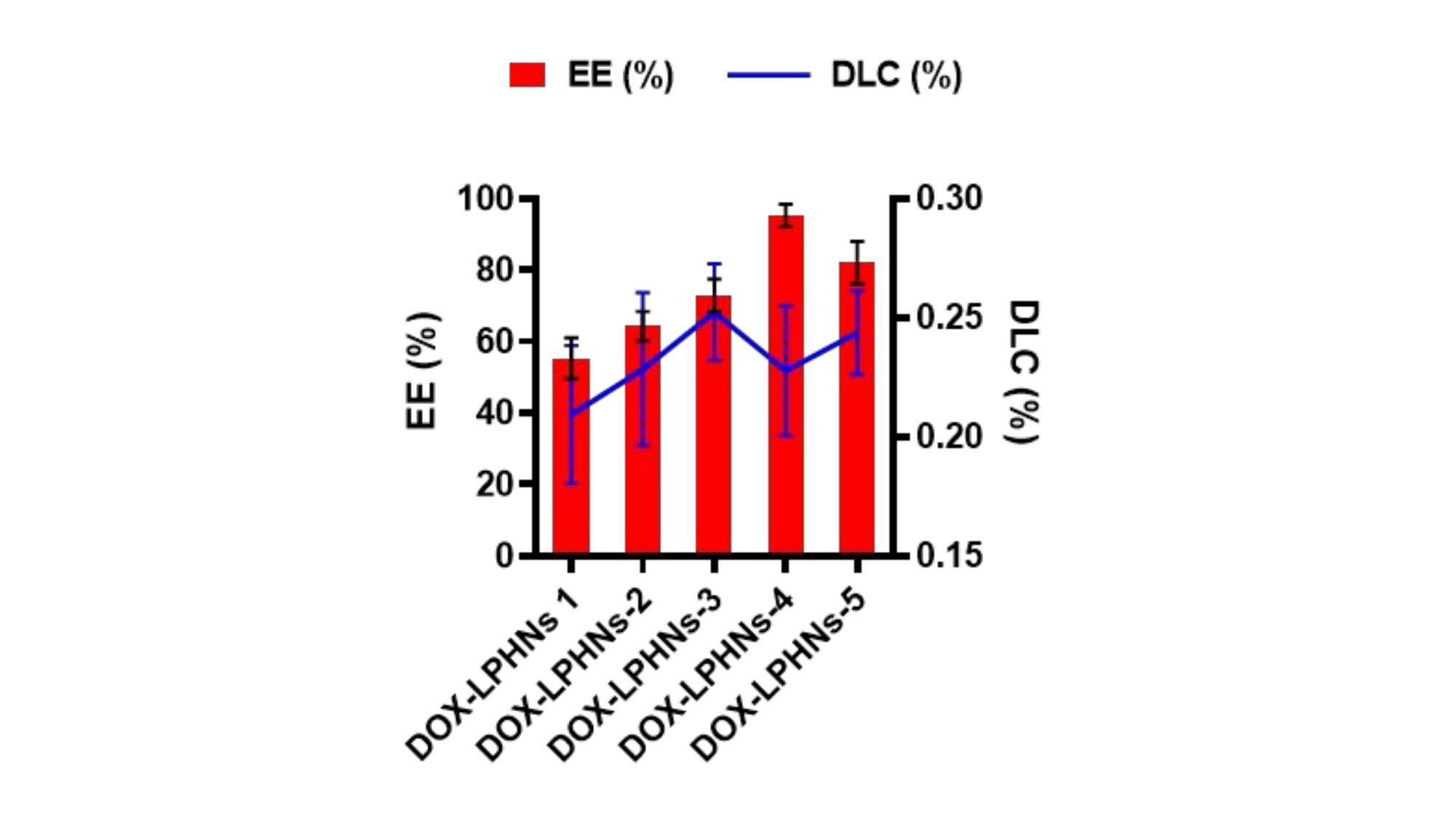

## Slide 4
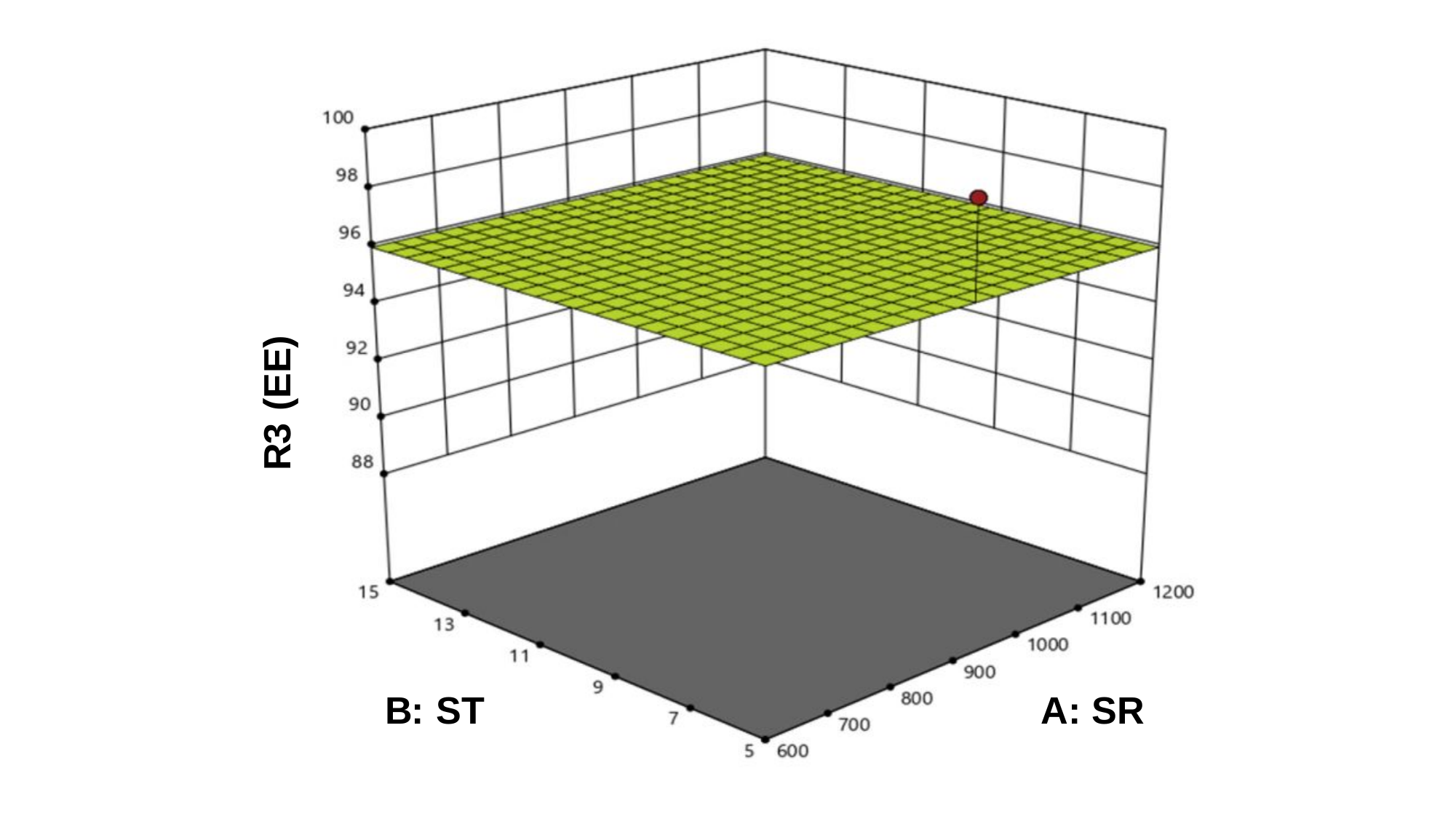

## Slide 5
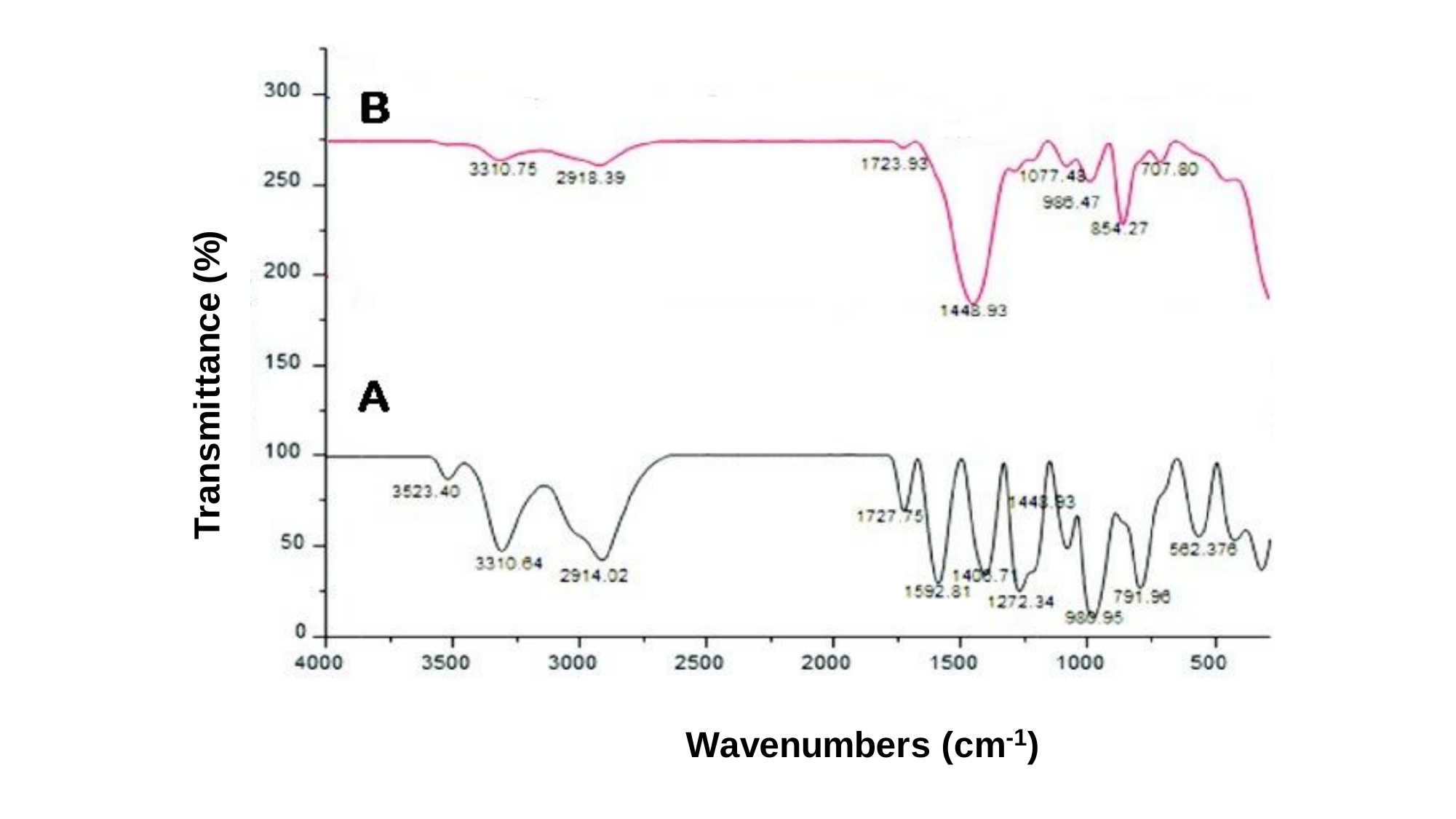

## Slide 6
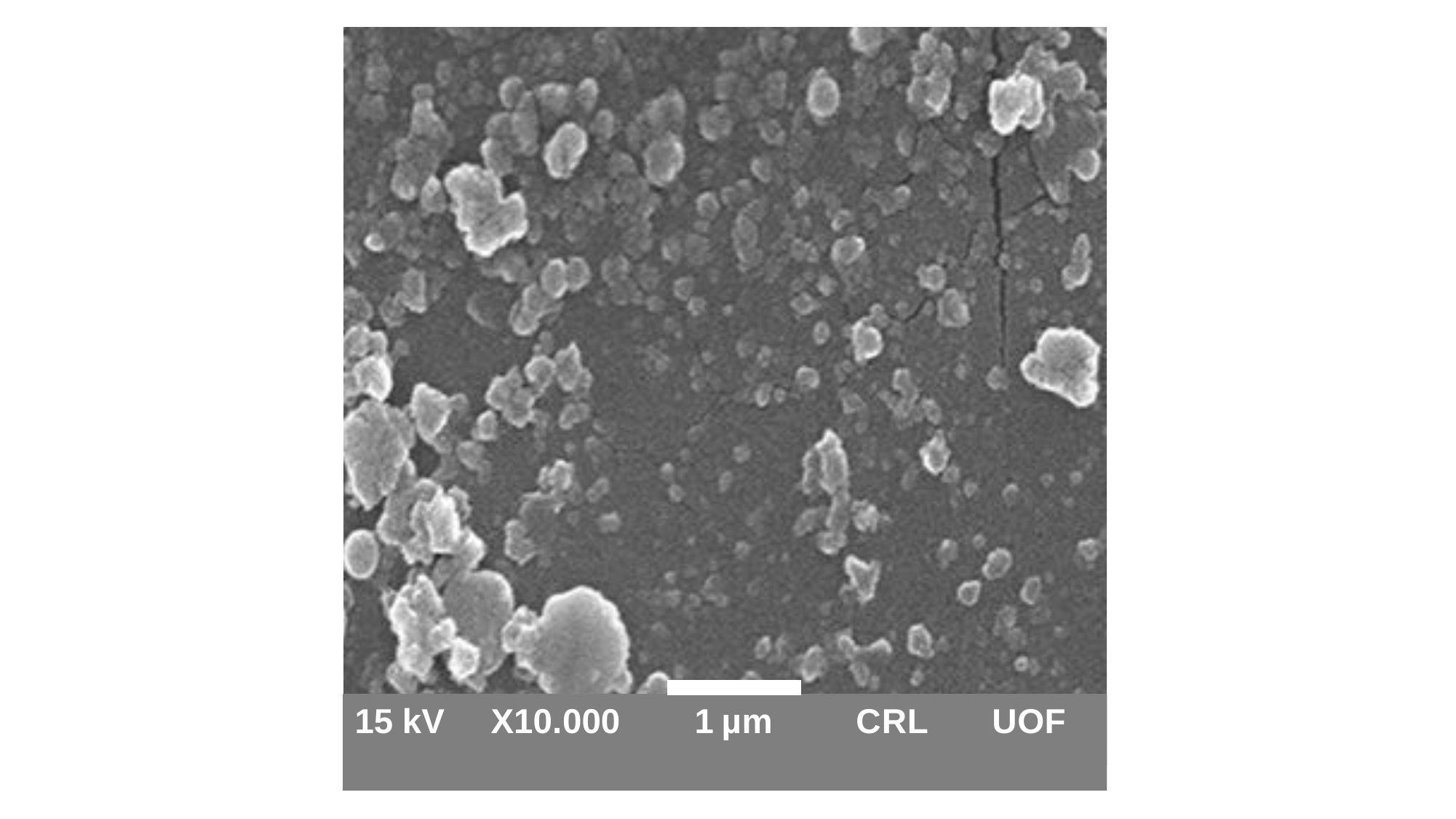

## Slide 7
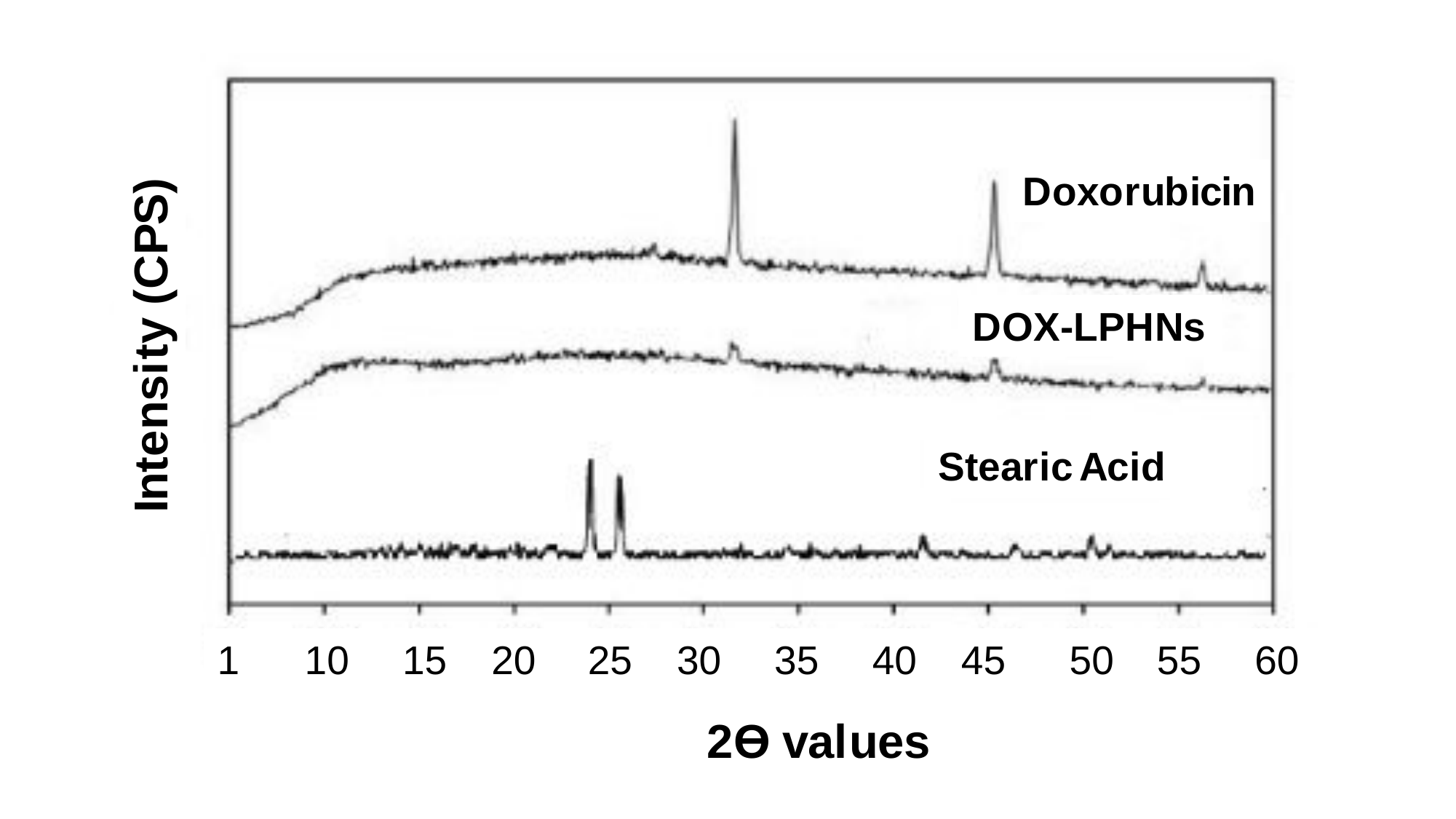

## Slide 8
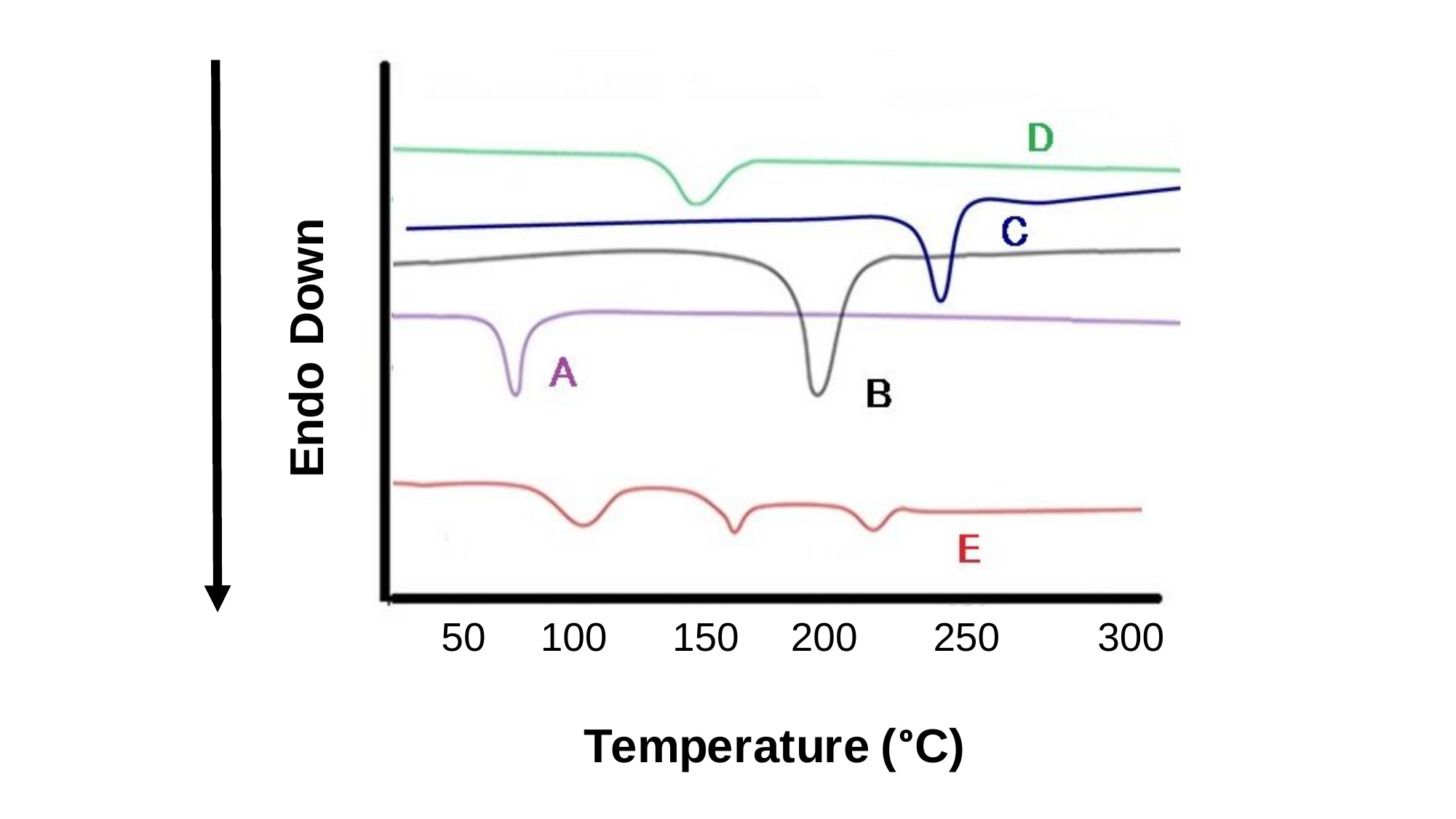

## Slide 9
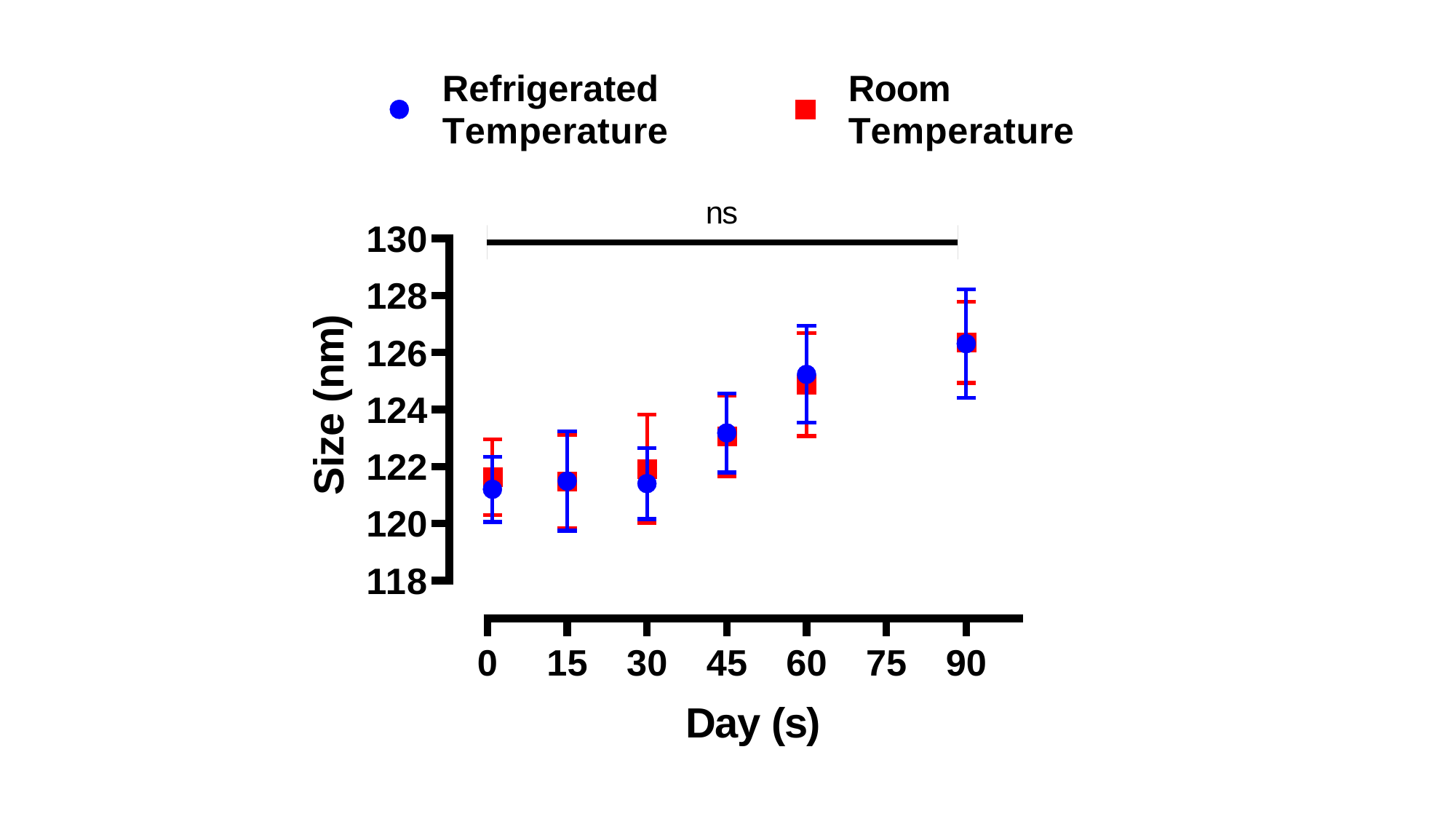

## Slide 10
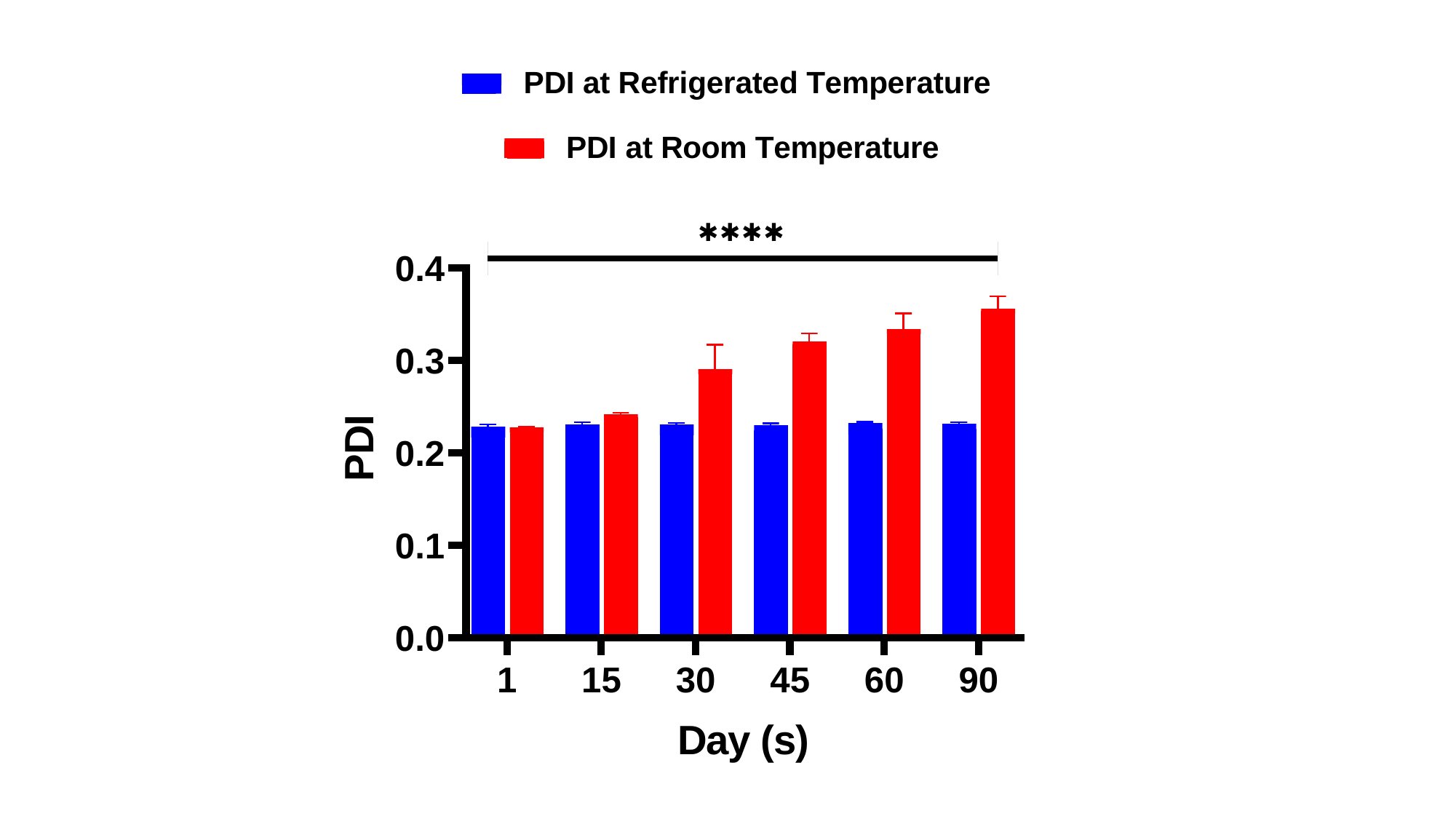

## Slide 11
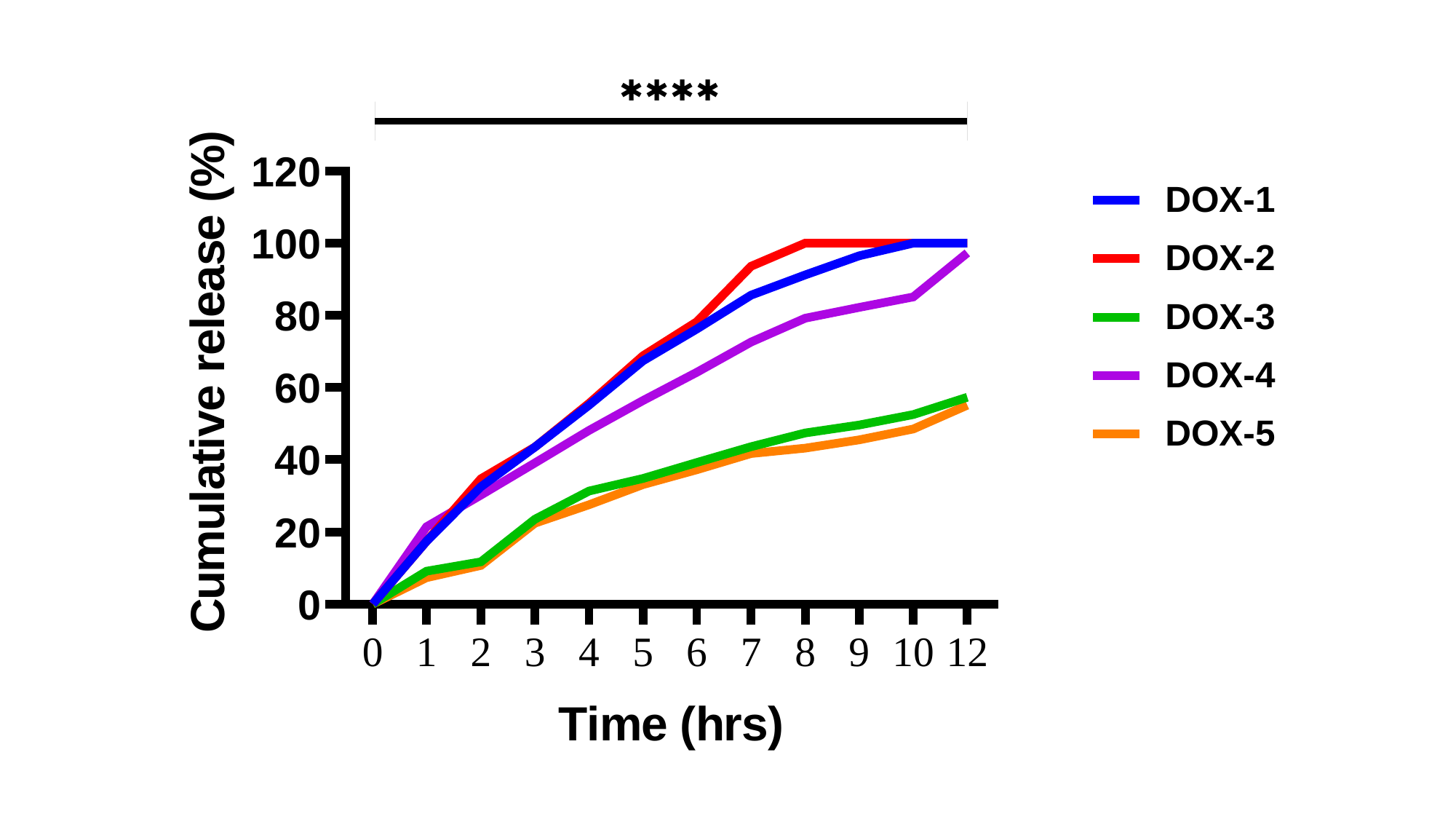

## Slide 12
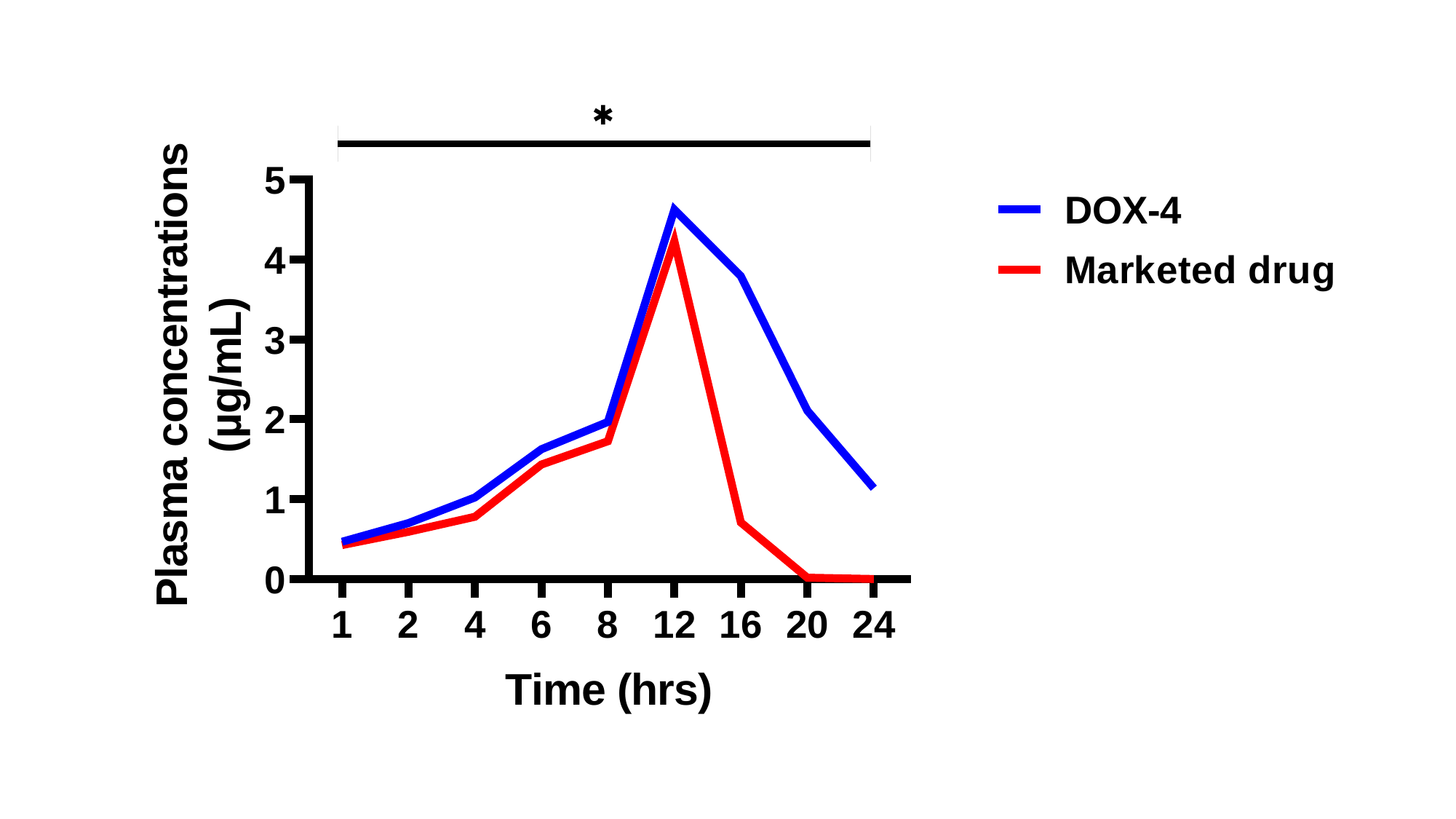

## Slide 13
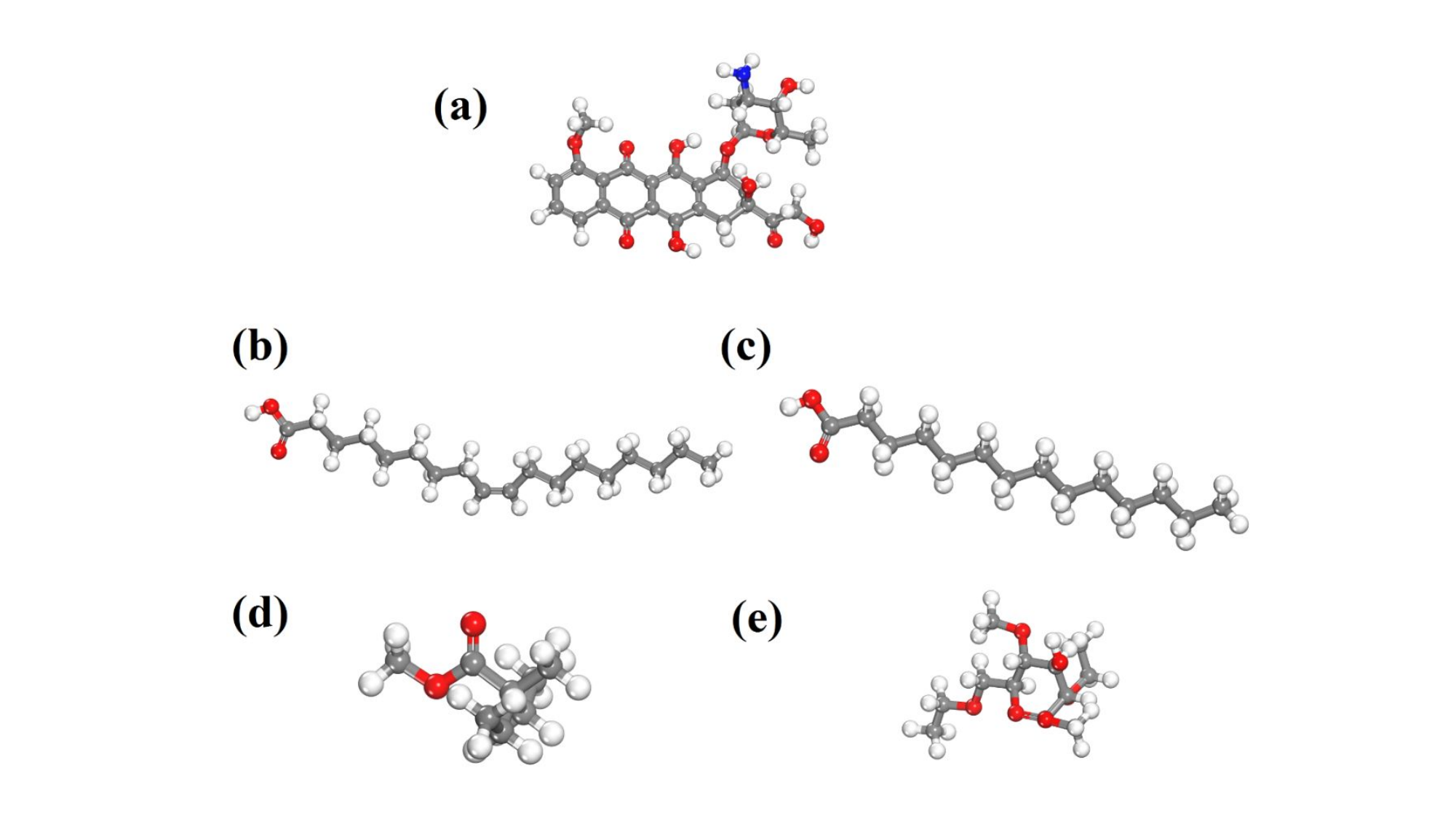

## Slide 14
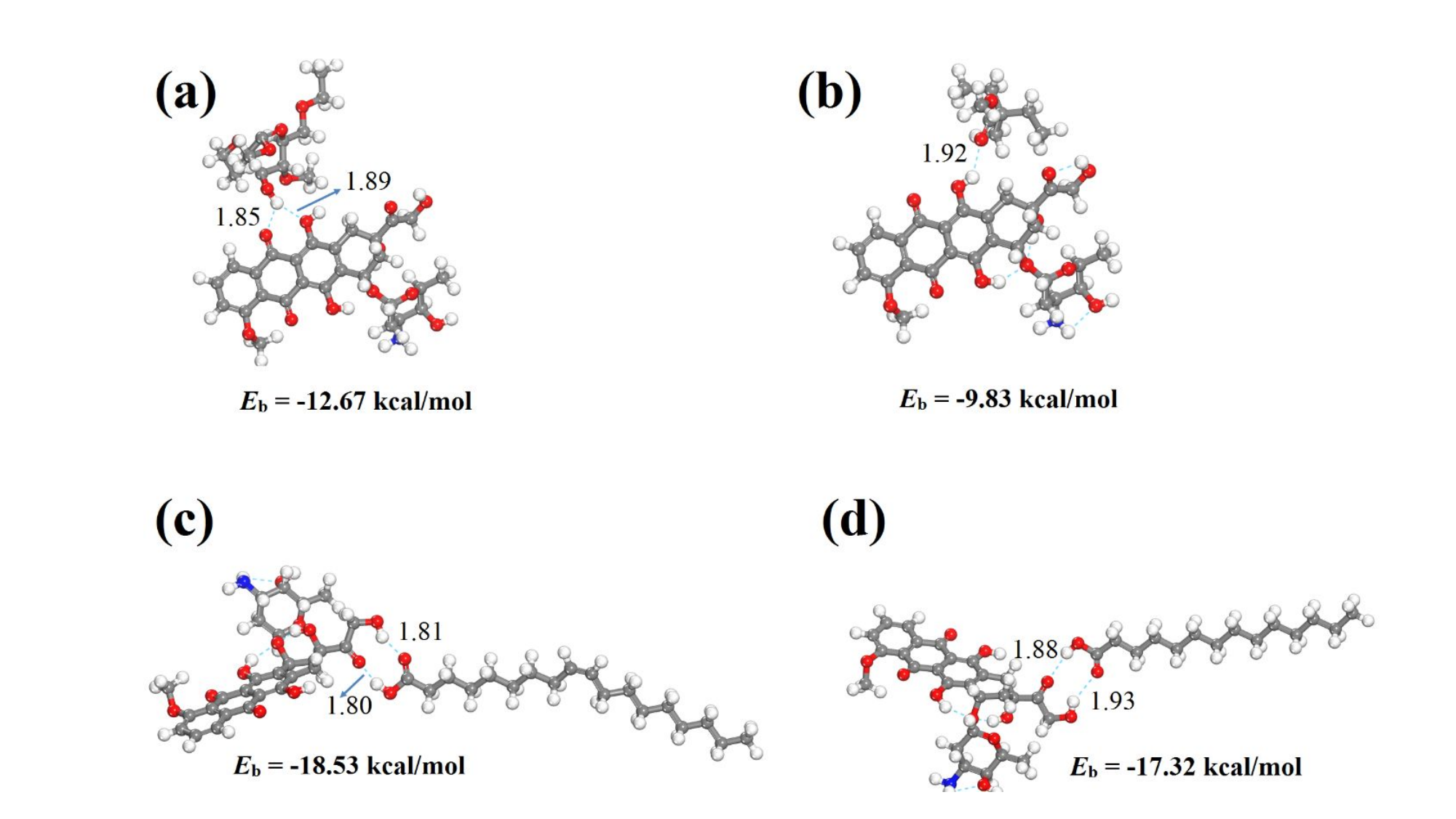

Supplement: Supplementary file 1 [file Presentation1.PPTX]
